# KSHV RTA antagonizes SMC5/6 complex-induced viral chromatin compaction by hijacking the ubiquitin-proteasome system

**DOI:** 10.1371/journal.ppat.1010744

**Published:** 2022-08-01

**Authors:** Chunyan Han, Dun Zhang, Chenwu Gui, Liang Huang, Sijia Chang, Lianghui Dong, Lei Bai, Shuwen Wu, Ke Lan

**Affiliations:** 1 State Key Laboratory of Virology, College of Life Sciences, Wuhan University, Wuhan, China; 2 Department of Infectious Diseases, Frontier Science Center for Immunology and Metabolism, Medical Research Institute, Zhongnan Hospital of Wuhan University, Wuhan University, Wuhan, China; 3 Taikang Center for Life and Medical Sciences, Wuhan University, Wuhan, China; University of North Carolina at Chapel Hill, UNITED STATES

## Abstract

Kaposi’s sarcoma-associated herpesvirus (KSHV) is a double-stranded DNA virus with the capacity to establish life-long latent infection. During latent infection, the viral genome persists as a circular episome that associates with cellular histones and exists as a nonintegrated minichromosome in the nucleus of infected cells. Chromatin structure and epigenetic programming are required for the proper control of viral gene expression and stable maintenance of viral DNA. However, there is still limited knowledge regarding how the host regulates the chromatin structure and maintenance of episomal DNA. Here, we found that the cellular protein structural maintenance of chromosome (SMC) complex SMC5/6 recognizes and associates with the KSHV genome to inhibit its replication. The SMC5/6 complex can bind to the KSHV genome and suppress KSHV gene transcription by condensing the viral chromatin and creating a repressive chromatin structure. Correspondingly, KSHV employs an antagonistic strategy by utilizing the viral protein RTA to degrade the SMC5/6 complex and antagonize the inhibitory effect of this complex on viral gene transcription. Interestingly, this antagonistic mechanism of RTA is evolutionarily conserved among γ-herpesviruses. Our work suggests that the SMC5/6 complex is a new host factor that restricts KSHV replication.

## Author summary

KSHV can establish life-long latent infection. During latency, the viral genome is maintained as an extrachromosomal episome in the infected cells. We demonstrated that the host protein SMC5/6 complex associates with the KSHV genome and results in direct transcriptional inhibition by creating a transcriptionally repressive chromatin structure of the viral genome. RTA, the master switch protein of KSHV, can hijack the ubiquitin-proteasome system to degrade the SMC5/6 complex to antagonize its inhibitory effect on viral gene transcription. Interestingly, this function of RTA is evolutionarily conserved among γ-herpesviruses.

## Introduction

Kaposi’s sarcoma-associated herpesvirus (KSHV), also known as human herpesvirus 8, is the major causative agent of several human malignancies, including Kaposi sarcoma (KS), primary effusion lymphoma (PEL), and multicentric Castleman’s diseases (MCDs) [1]. Like other herpesviruses, KSHV employs two replication programs, the default latent replication and lytic replication [2]. Latent infection is characterized by no detectable production of infectious viral particles and expression of only a limited number of viral genes, including those encoding ORF73 (latency-associated nuclear antigen, LANA), ORF72 (viral Cyclin, vCyclin), ORF71 (viral Fas-associated death domain-like IL-1-converting enzyme inhibitory protein, vFLIP), kaposins, polyadenylated nuclear RNA (PAN RNA) and 25 mature microRNAs (miRNAs) [3]. During latency, multiple copies of the viral genome are maintained as circular episomes in the nucleus of infected cells, which are regulated by host epigenetic factors, either by promoting constitutive expression of latent genes or by blocking the expression of lytic genes [4,5]. Epigenetic programming of the viral episome is important for the virus life cycle, including the proper regulation of viral transcription during latency, cell cycle-regulated DNA replication, protection and repair of the viral genome, and the ability to switch to lytic replication. LANA is a dedicated episome maintenance protein (EMP) that plays a key role in viral episome maintenance and epigenetic programming. To date, studies have suggested that KSHV episomal DNA is clearly tethered to cellular chromosomes by LANA and segregates evenly to daughter cells during cell division. LANA binds to the terminal repeats (TRs) of the viral genome through its carboxy-terminus and tethers the viral genome to the host chromatin through its amino-terminus via interaction with histones [6–8]. The virus can emerge from latency in response to various intracellular or extracellular stimuli, such as valproic acid (VPA), hypoxia, 12-O-tetradecanoylphorbol-13-acetate (TPA) and butyrate [9]. During the lytic phase, all viral genes are expressed in a cascading fashion, and infectious virus particles are generated. Genes expressed during the lytic cycle can be classified as immediate-early (IE), early (E) and late (L) genes according to their timing of expression in response to protein synthesis or DNA replication inhibitors [10,11]. Replication and transcription activator (RTA), an immediate-early gene encoded by ORF50, is the switch protein for reactivation of KSHV from latency. In addition to transcription activation, RTA can function as a ubiquitin E3 ligase. It has been shown that RTA can specifically target IRF7, MyD88, TRIF, NF-κB and HLA-DRα for proteasomal degradation as a mechanism to antagonize innate immunity [12–14]. In addition, RTA can also recruit and stabilize the cellular ubiquitin ligase RAUL to target IRF3 and IRF7 for proteasomal degradation [15]. Aside from its role in modulating the innate immune response, RTA can also degrade a number of repressors such as Hairy/enhancer-of-split related to YRPW motif protein 1 (Hey1), KSHV-RTA binding protein (K-RBP), as well as viral proteins such as LANA, K8 and vFLIP [16–18]. However, the number of proteins degraded by RTA far exceed those identified here.

The SMC5/6 complex, together with cohesion and condensin, is a member of the ring-shaped structural maintenance of chromosome (SMC) complex found in eukaryotes [19,20]. The SMC5/6 complex contains two core proteins, SMC5 and SMC6, and four regulatory subunits, referred to as non-SMC elements (NSE1 to 4). Structurally, the SMC5 and SMC6 heterodimers are folded into an unclosed ring-like structure containing an ATPase head domain at one end (“head”), an intramolecular coiled-coil region (“arm”), and a hinge domain at the other end (“hinge”). The non-SMC subunit kleisin bridges the head domains of the SMC proteins to form a ring-like structure. Additional components are docked into the core scaffold to support and provide specific functions. Functionally, the SMC5/6 complex is best known for DNA repair and sister chromatid recombination, and genome stability [21–23]. However, recent work shows that the role of SMC5/6 goes far beyond DNA repair and genome stability. A potential role in viral infection has been suggested for the SMC5/6 complex, including for hepatitis B virus (HBV), human papillomavirus (HPV) and HIV-1 [24–27]. The SMC5/6 complex is considered to be a restriction factor for HBV and HIV-1. During HBV infection, the SMC5/6 complex induces the repression of extrachromosomal covalently closed circular DNA (cccDNA) transcription [25]. During HIV-1 infection, SMC5-SMC6 complex localization factor 2 (SLF2) was identified as a Vpr target and is responsible for silencing the transcription of unintegrated HIV-1 DNA [26]. However, the detailed mechanism of how transcription is suppressed is unclear. Interestingly, during HPV infection, the SMC5/6 complex does not affect E2 or other viral gene transcriptional activation, suggesting different roles of SMC5/6 in HBV, HIV-1 and HPV infections [24].

In the present study, we demonstrated that the SMC5/6 complex is a novel binding protein of the KSHV episome that can recognize and associate with KSHV genomic DNA and pose a significant inhibitory effect on viral replication. In detail, the SMC5/6 complex suppresses KSHV gene transcription by condensing viral chromatin and creating a repressive chromatin structure. However, KSHV evolves an equally potent counterrestriction mechanism by utilizing RTA to degrade the SMC5/6 complex, and this antagonistic mechanism is evolutionarily conserved among γ-herpesviruses. This evidence strongly suggests that the SMC5/6 complex is a new host factor that can restrict the infection of herpesviruses.

## Results

### Ectopic expression of SMC5 and SMC6 inhibits KSHV lytic replication

**Fig 1:**
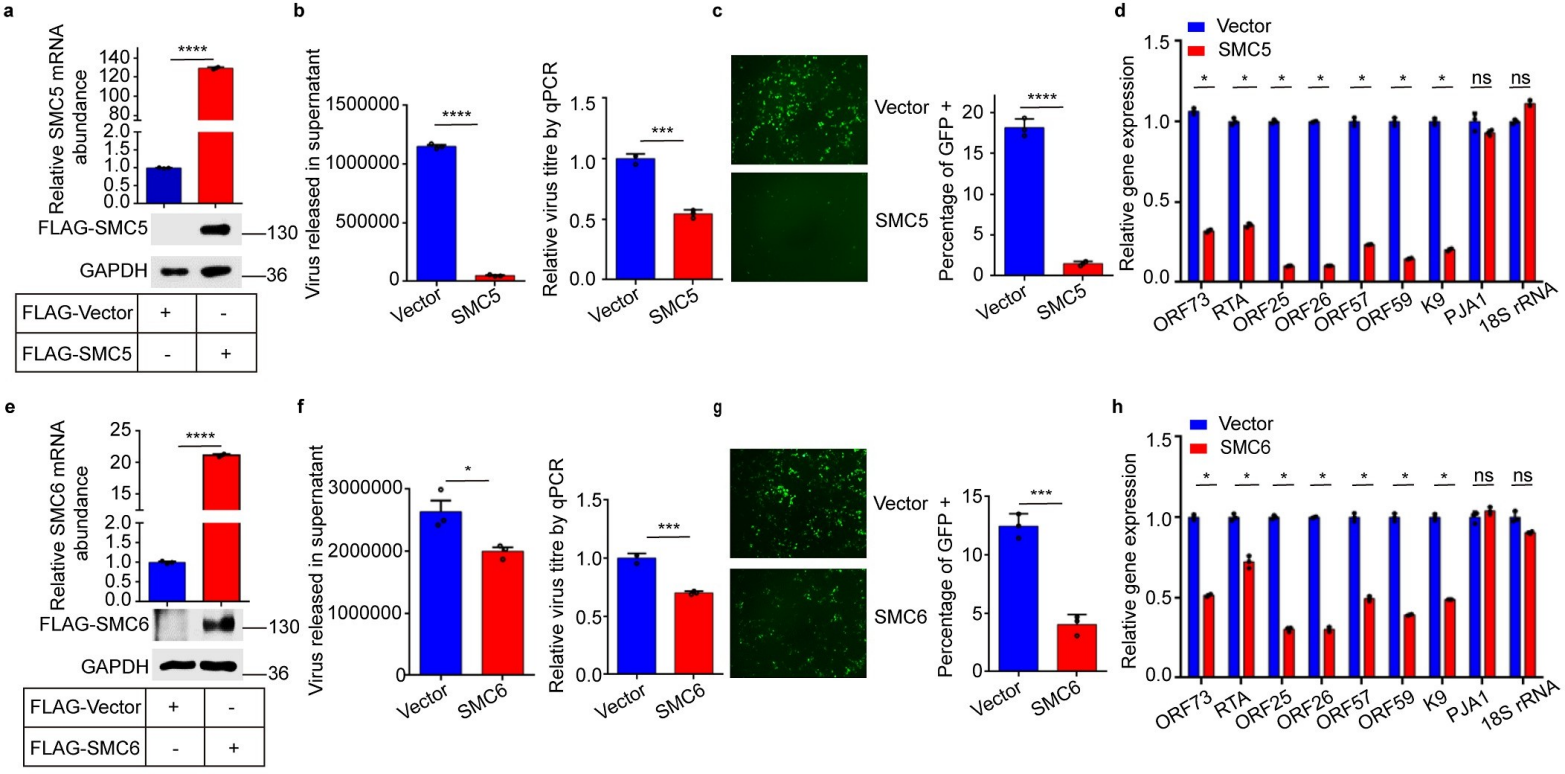
Ectopic expression of SMC5 and SMC6 inhibits KSHV lytic replication. (a) The expression of SMC5 was evaluated via qPCR and immunoblot. (b) DNase-treated extracellular viral loads of SMC5 were quantified via qPCR. Left panel, DNA copy numbers of extracellular viruses were quantified by using K9 primers. Right panel, after DNase treatment, pGL3-luc plasmid DNA was added during viral DNAs extraction to ensure the quality of DNA extraction. Relative DNA copy numbers were measured via qPCR using primers for K9 and pGL3. The values of control were set as 1. (c) Cell supernatants of SMC5-overexpressing cells were collected and infected with HEK293T cells. 24h later, KSHV-infected GFP-positive cells were observed by fluorescence microscope and analyzed by flow cytometry. (d) Viral genes and cellular genes expression were analyzed via qPCR. (e-h) Effect of SMC6 on KSHV lytic replication, which is similar to (a-d). Representative results from three biological replicates are presented. Error bars indicate SD. Data were analyzed with Student’s multiple t tests (*p < 0.05, ***p< 0.001, ****p< 0.0001).

To determine whether the SMC5/6 complex has an impact on KSHV replication, we overexpressed SMC5 or SMC6 in iSLK.RGB cell line, which was stably infected with a reporter virus [28]. SMC5 and SMC6 mRNA were abundantly expressed in SMC5- and SMC6-overexpressing cells (Fig 1A and 1E). Consistent with the mRNA levels, SMC5 and SMC6 proteins were detected in overexpressing cells using FLAG tag antibody (Fig 1A and 1E) or antibodies against SMC5 or SMC6 (S1A and S1B Fig). The results showed that ectopic expression of SMC5 enhanced endogenous protein levels of SMC6, and overexpression of SMC6 also enhanced endogenous protein levels of SMC5. To induce lytic replication of KSHV, these cell lines were treated with doxycycline (Dox) and sodium butyrate (NaB). As shown in Fig 1B and 1F, we found that overexpression of SMC5 and SMC6 reduced the DNase-treated extracellular viral loads by ~90% and ~30%, respectively. To further confirm the effect of SMC5/6 on the infectivity of progeny viruses, we utilized the cell supernatants to reinfect naïve HEK293T cell monolayers. First, we observed a significant decrease in green fluorescence in HEK293T cells infected with viral supernatants of SMC5- and SMC6 overexpressing cells (Fig 1C and 1G, left panels). Then, we quantified the percentage of GFP-positive cells by flow cytometry and found that the percentage of GFP-positive cells in HEK293T cells infected with viral supernatants of SMC5- or SMC6- overexpressing cells was decreased by ~90% or ~60% (Fig 1C and 1G, right panels). Moreover, we analyzed the expression of latent, immediate-early, early and late genes via qPCR and found that the expression of both the lytic and the latent genes of KSHV decreased by ~60%-80% and ~30%-60% in the SMC5 and SMC6 stable expression cell lines, which was similar to the results of the viral loads (Fig 1D and 1H), indicating that ectopic expression of SMC5 and SMC6 decreases KSHV replication.

### Knockdown of SMC5 and SMC6 promotes KSHV lytic replication

**Fig 2:**
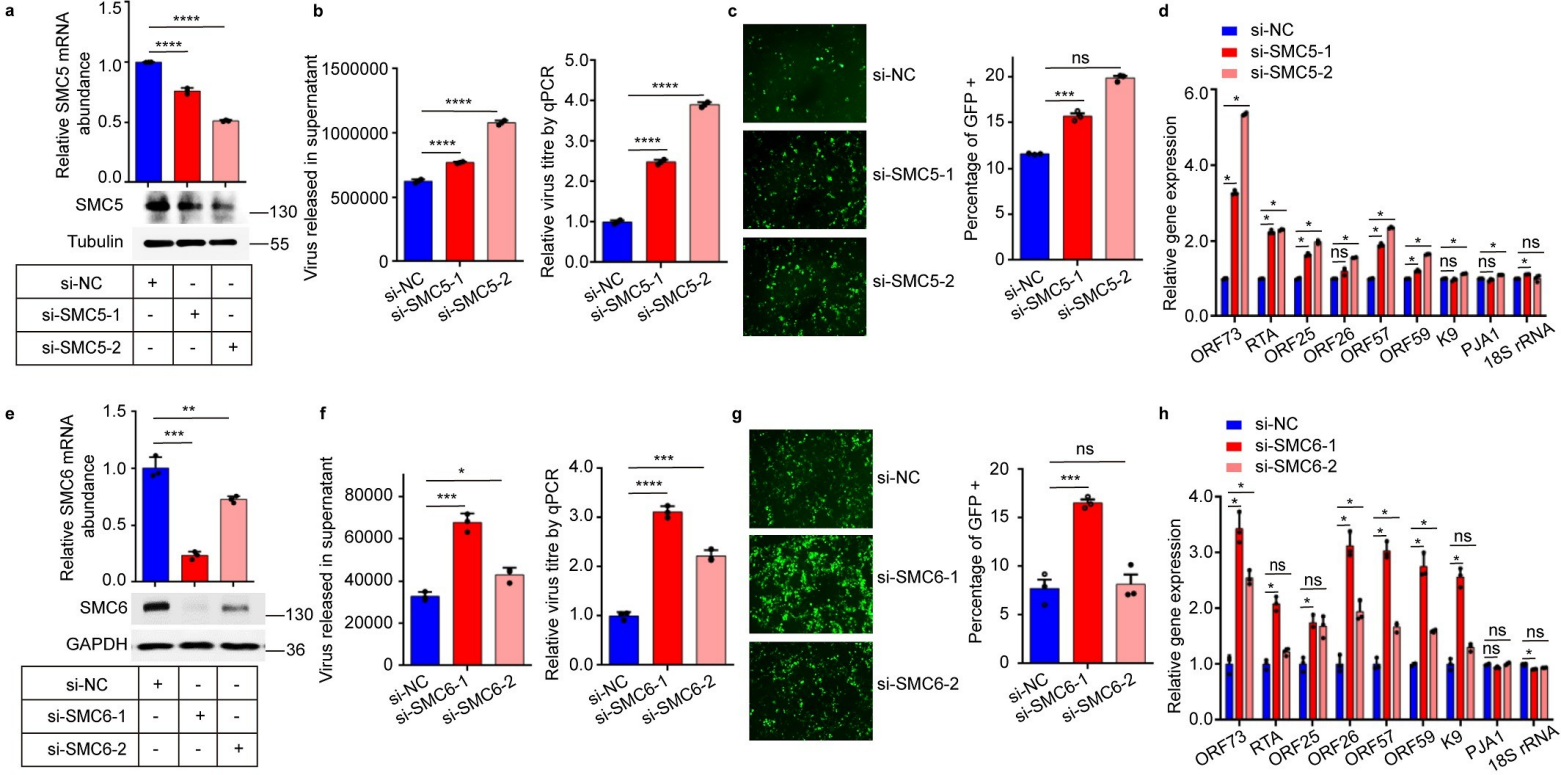
Knockdown of SMC5 and SMC6 promotes KSHV lytic replication. (a) iSLK.RGB cells were transfected with control siRNA and two siRNAs targeting human SMC5. 48h post-transfection, the knockdown efficiency was determined via qPCR and western blots. (b) DNase-treated viral DNAs from culture supernatants were analyzed via qPCR. Left panel, viral DNA copy numbers were quantified by using K9 primers. Right panel, pGL3-luc plasmid DNA was added during viral DNAs extraction to ensure the quality of DNA extraction. Relative DNA copy numbers were measured via qPCR using primers for K9 and pGL3. The values of control were set as 1. (c) Cell supernatants of SMC5-knockdown cells were collected and infected with HEK293T cells. 24h later, KSHV-infected GFP-positive cells were observed by fluorescence microscope and analyzed by flow cytometry. (d) SMC5-knockdown and control cells were treated with Dox and NaB for 48h, and KSHV gene transcription was analyzed via qPCR. (e-h) Effect of knockdown SMC6 on KSHV lytic replication, which is similar to (a-d). Representative results from three biological replicates are presented. Error bars indicate SD. Data were analyzed with Student’s multiple t tests (*p < 0.05, **p < 0.01, ***p< 0.001, ****p< 0.0001).

To further confirm the effect of the SMC5/6 complex on KSHV lytic replication, we transfected two siRNAs targeting human SMC5 and SMC6, respectively, in iSLK.RGB cells to deplete the expression of SMC5 and SMC6 before induction with Dox and NaB. The knockdown efficiency was measured via qPCR and western blots, and the results showed that the two siRNAs against SMC5 reduced the SMC5 protein levels by 31% and 68% compared with that of the nontargeting control (NC) (Fig 2A). Likewise, siRNAs against SMC6 reduced the protein levels of SMC6 by 90% and 57% (Fig 2E). Importantly, we also found that knockdown of SMC5 and SMC6 reduced the expression of endogenous SMC6 and endogenous SMC5, respectively (S1C and S1D Fig).

As shown in Fig 2B and 2C, the DNase-treated extracellular viral load in SMC5-knockdown cells was ~2-fold higher than that in the NC group, and the percentage of GFP-positive cells in HEK293T cells infected with the viral supernatant of SMC5-knockdown cells increased by ~2-fold. Similarly, knockdown of SMC6 increased the DNase-treated extracellular viral load and the percentage of GFP-positive cells in HEK293T cells by ~2-fold (Fig 2F and 2G). In addition, the expression of both the lytic-related and the latent genes of KSHV was ~1.5–3.4-fold higher than that in the NC group (Fig 2D and 2H). Moreover, we depleted SMC5 or SMC6 in KSHV-infected BCBL1 cells and detected the DNase-treated extracellular viral load. The results showed that knockdown of SMC5 or SMC6 in BCBL1 cells promoted KSHV replication (S2 Fig). Taken together, these results convincingly suggest that knockdown of SMC5 or SMC6 effectively enhances KSHV replication.

### The SMC5/6 complex associates with the KSHV genome and suppresses KSHV gene transcription

**Fig 3:**
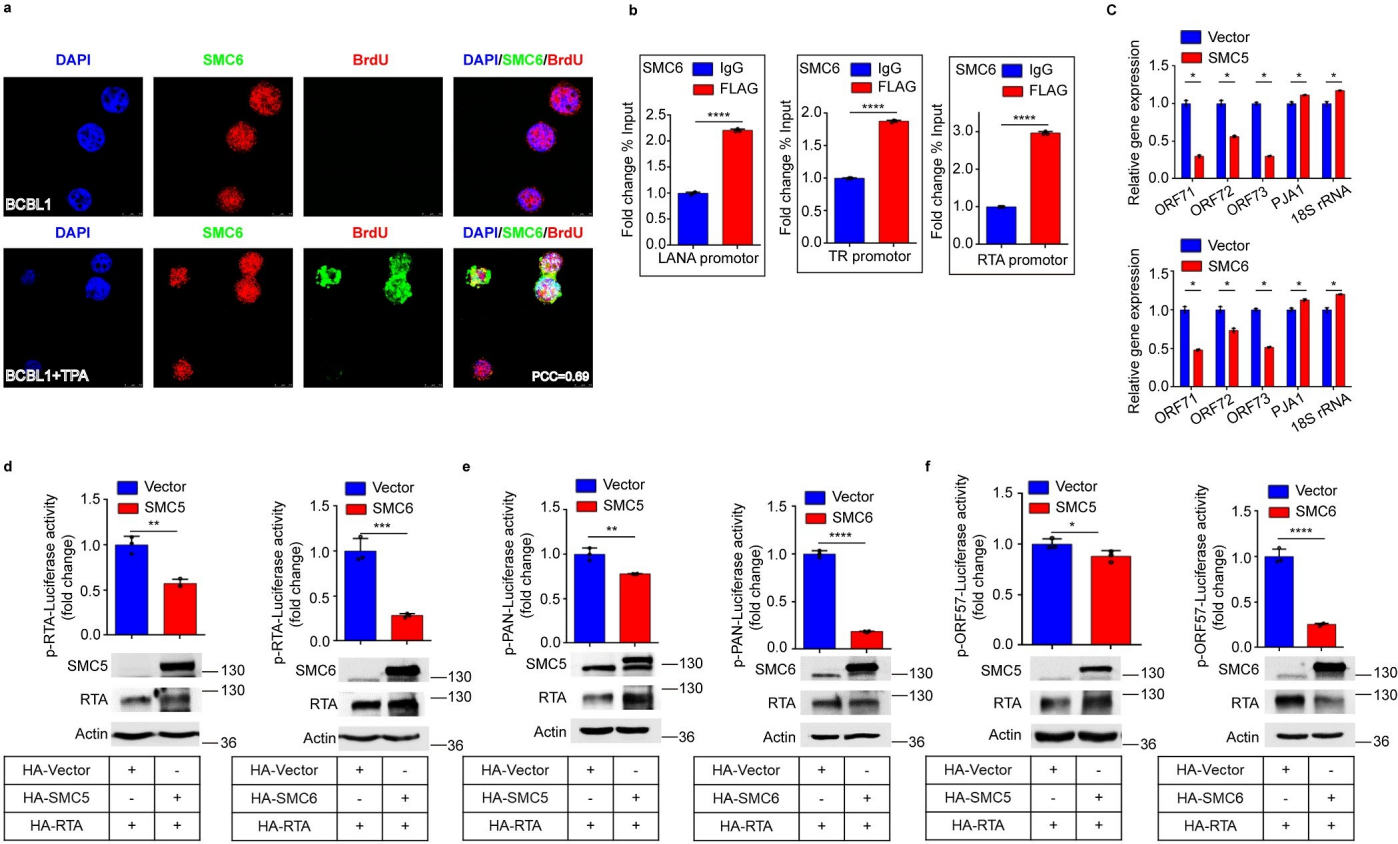
The SMC5/6 complex associates with the KSHV genome and suppresses KSHV gene transcription. (a) The SMC5/6 complex colocalizes with KSHV genomic DNA. BCBL1 cells were treated with TPA (20ng/ml) for 24h followed by incubating with BrdU for 2h, then immunofluorescence was performed to observe the colocalization of endogenous SMC6 and KSHV genomic DNA. Colocalization analysis was performed using Image J software. (b) SMC6 binds to the KSHV genome. ChIP experiments were performed in latently infected SMC6-overexpressing cells using anti-FLAG antibody and the episomal DNA of KSHV was measured by qPCR. Data was calculated as the fold change in percentage of input DNA compared with control ChIP experiment. (c) Effect of SMC5 and SMC6 on KSHV latent genes transcription (ORF71, ORF72 and ORF73). Total RNA extracted from latently infected SMC5- and SMC6-overexpressing cells was evaluated via qPCR. (d-f) Effect of SMC5 and SMC6 on RTA-mediated RTA, PAN and ORF57 promoter activation. HEK293T cells were transfected with 200ng RTA/PAN/ORF57-promoter and other plasmid DNA as indicated. 36h post-transfection, cells were lysed and luciferase activities were analyzed. Lysates of cells were also subjected to western blot analysis. Representative results from three biological replicates are presented. Error bars indicate SD. Data were analyzed with Student’s t tests (*p < 0.05, **p< 0.01, ***p< 0.001, ****p< 0.0001).

Previously, it was shown that the SMC5/6 complex can bind to DNA and plays an important role in chromosome dynamics and stability [29]. To determine whether SMC6 can bind to KSHV genomic DNA, we utilized BrdU to label the newly synthesized KSHV genome [30]. To avoid BrdU incorporation into the cellular genome, the cells were fixed and stained with anti-BrdU antibody and anti-SMC6 antibody 2 hours post-BrdU labeling. As shown in Fig 3A, SMC6 was predominantly localized in the cell nuclei in both BCBL1 cells and TPA-treated BCBL1 cells. Without TPA treatment, KSHV lytic replication could not be reactivated, and BrdU could not be detected. However, in TPA-treated BCBL1 cells, BrdU-labeled viral DNA was also observed in the nucleus, and most of it colocalized with SMC6. The results of colocalization analysis showed that the Pearson’s correlation coefficient (PCC) was 0.69, indicating that BrdU-labeled viral DNA colocalized with SMC6. Importantly, we also performed chromatin immunoprecipitation (ChIP) in iSLK.RGB cells to validate the association of SMC6 with the KSHV genome. ChIP assays were performed by using FLAG antibody or IgG antibody in latently infected SMC6- overexpressing cells. As show in Fig 3B, the KSHV genome could be enriched by FLAG antibody but not the IgG control, indicating that SMC6 can bind to the KSHV genome. While our data showed that SMC5/6 can bind to the KSHV genome and inhibit KSHV replication, we propose that the SMC5/6 complex may directly block viral gene expression of KSHV. To test this hypothesis, we measured the mRNA expression of the latent genes ORF71, ORF72 and ORF73 in SMC5 or SMC6 stable expression cell lines via qPCR and found that overexpression of SMC5 and SMC6 significantly decreased the expression of latent genes by ~30%-70% (Fig 3C). Interestingly, during lytic replication, overexpression of SMC5 and SMC6 inhibited the expression of immediate-early, early and late genes. This indicated that the inhibitory effect of the SMC5/6 complex on KSHV gene transcription is not gene specific, but a pan effect may result from chromatin structure remodeling. To corroborate the above result, we also investigated whether the SMC5/6 complex is involved in regulating the gene expression of KSHV during the lytic cycle by using a luciferase reporter gene assay. The results showed that SMC5 and SMC6 suppressed RTA-mediated RTA, PAN, and ORF57 promoter activation (Fig 3D–3F).

### The SMC5/6 complex suppresses gene transcription in ATPase- and DNA-binding activity-dependent manners

**Fig 4:**
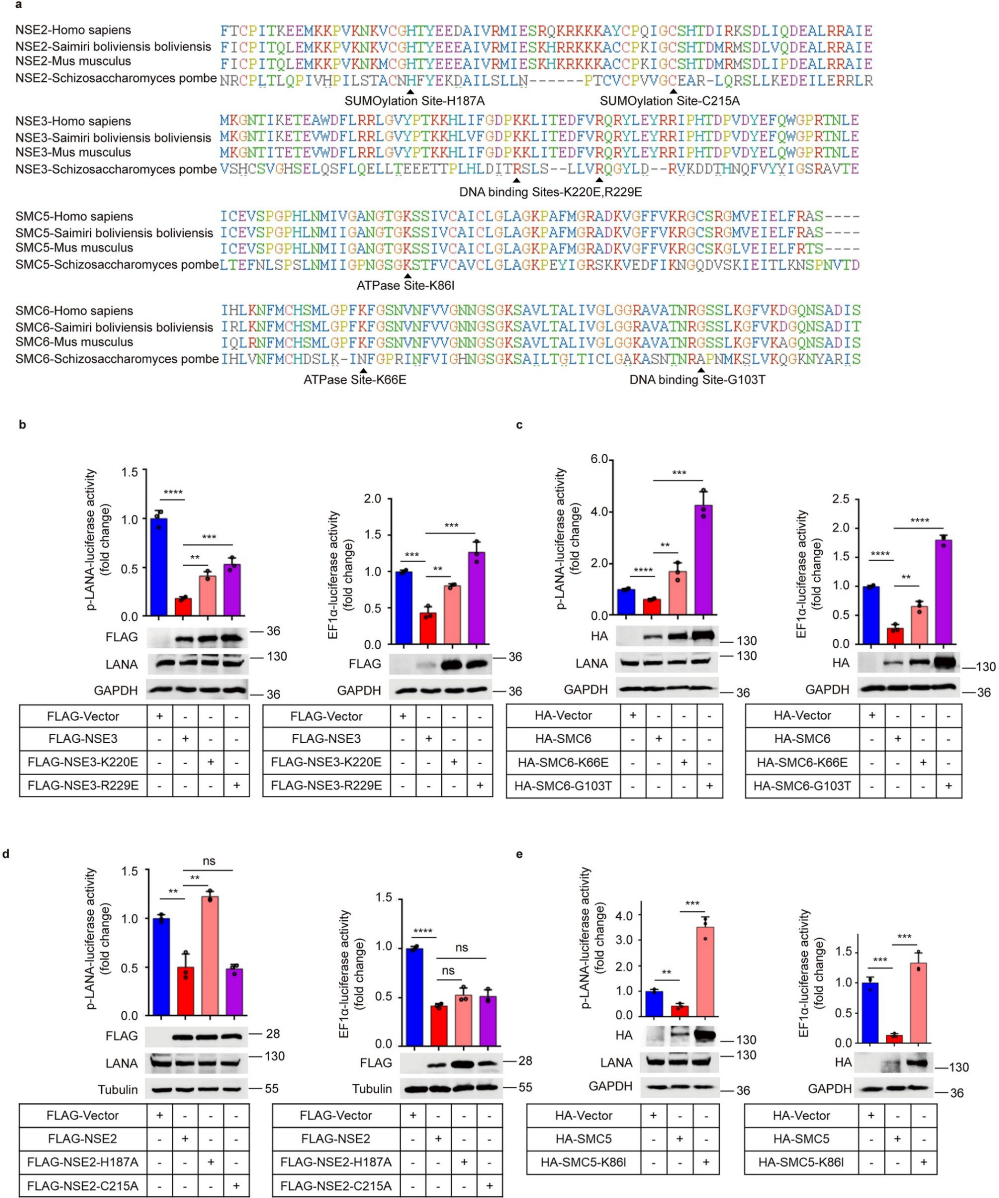
The SMC5/6 complex suppresses gene transcription in ATPase- and DNA-binding activity-dependent manners. (a) Schematic diagram of DNA-binding, SUMOylation and ATPase mutations of the SMC5/6 complex. Mutate the corresponding active sites of *homo sapiens* according to the site mutations of yeast. (b) Effect of DNA binding-defective mutates of NSE3 on KSHV gene transcription. HEK293T cells were transfected with 200ng LANA-promoter (left) or EF1αpromotor (right), inactive mutations and other plasmid DNA as indicated. 36h post-transfection, cells were lysed and luciferase activities were analyzed. In addition, lysates of cells were subjected to western blot analysis. (c) Effect of inactive DNA-binding mutation and ATPase mutation of SMC6 on KSHV gene transcription. (d) Effect of inactive SUMOylation mutations of NSE2 on KSHV gene transcription. (e) Effect of inactive ATPase mutation of SMC5 on KSHV gene transcription. Representative results from three biological replicates are presented. Error bars indicate SD. Data were analyzed with Student’s multiple t tests (**p< 0.01, ***p< 0.001, ****p< 0.0001).

The results above suggest that the SMC5/6 complex binds to KSHV genomic DNA and inhibits KSHV gene transcription. It has been demonstrated that all SMC complexes share a common property, which is to organize chromosomes by topologically embracing DNA inside their ring-shaped structure [31]. To explore the mechanism by which the SMC5/6 complex suppresses KSHV DNA transcription, we focused on the chromosome dynamics and organization functions of the SMC5/6 complex. It has been reported that SMC5/6 can bind to DNA through different DNA-binding interfaces: the hinge, arms and ATPase head regions of SMC5 and SMC6 proteins, and the surface of the NSE1-NSE3 subcomplex. SMC5/6 and NSE1-NSE3 might be the initial DNA contact points [32–34]. By aligning the sequences of *Homo sapiens*, *Mus musculus*, *Saimiri boliviensis boliviensis* and *Schizosaccharomyces pombe*, we mutated the corresponding active sites of *Homo sapiens* according to sites reported in yeast (Fig 4A). To compare the transcription-inhibiting activity between wild -type (WT) SMC5/6 and mutant SMC5/6, we cotransfected DNA binding-defective mutants of NSE3 and SMC6 or WT with the KSHV LANA promoter or cellular EF1α promoter and detected the activation of promoters using a luciferase assay. In comparison with WTs, K220E mutation of NSE3, R229E mutation of NSE3, and G103T mutation of SMC6 increased the activities of the LANA promoter and EF1α promoter by ~2-fold, ~3-fold and ~7-fold, respectively (Fig 4B and 4C). This suggests that an intact SMC5/6 complex is required for efficient loading onto episomal DNA. After loading onto DNA, direct binding of DNA to SMC5 is sufficient for NSE2-dependent SUMOylation [35]. The inactive SUMOylation mutations H187A and C215A of NSE2 render human cells sensitive to DNA damage, highlighting their relevance in genome maintenance [36]. By constructing SUMOylation inactive mutations and using a reporter assay, we found that mutations H187A and C215A had no effect on the activation of the EF1α promoter, while the mutation H187A increased the activity of the LANA promoter ~4-fold compared with that of the WT (Fig 4D). This suggests that SUMOylation activity may not be necessary for transcription silencing. More importantly, topologically embracing DNA requires ATPase activity [29]. Therefore, we evaluated the effect of ATPase inactive mutations (the mutations K86I of SMC5 and K66E of SMC6) in regulating KSHV transcription. We found that the activities of the LANA promoter and EF1α promoter in the ATPase mutation group were ~4-fold higher than those in the WT group and approximately 1.5-fold higher than those in the control group (Fig 4C and 4E). Hence, we concluded that the SMC5/6 complex associates with KSHV genomic DNA and suppresses KSHV gene transcription in ATPase- and DNA-binding activity-dependent manners, revealing that the SMC5/6 complex topologically captures KSHV episomes via energy produced by ATP hydrolysis.

### The SMC5/6 complex leads to a loss of H3K27ac on KSHV chromatin

**Fig 5:**
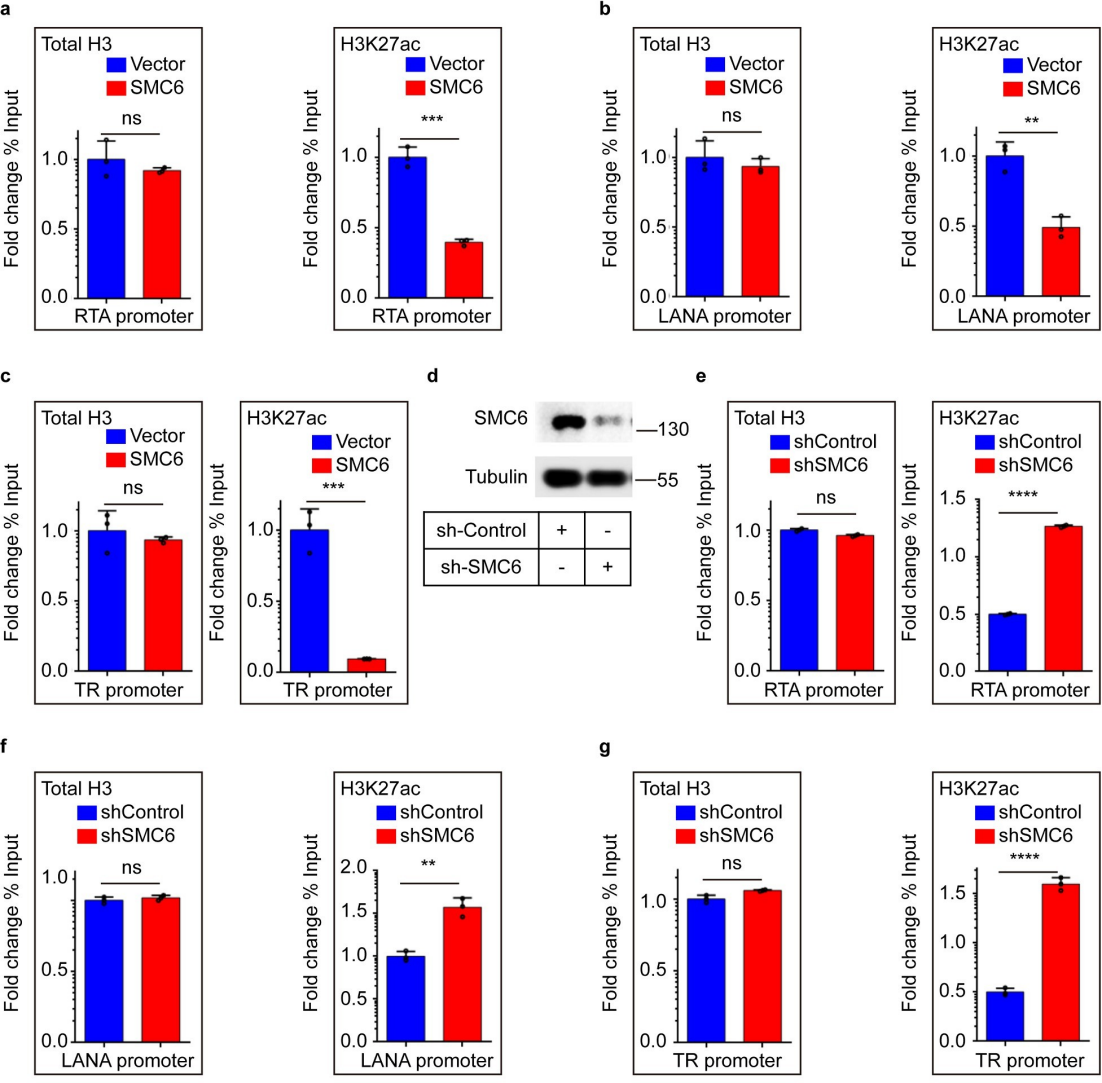
The SMC5/6 complex leads to a loss of H3K27ac on KSHV chromatin. (a-c) Ectopic expression of SMC6 decreases the levels of H3K27ac on KSHV genome. (a) H3K27ac on viral RTA was measured with ChIP-qPCR assay. ChIP was performed among latently infected control cells and SMC6-overexpressing cells by using antibodies against total H3 or H3K27ac. The recruitment of H3 and H3K27ac on KSHV RTA were tested via qPCR. Data was calculated as the fold change in percentage of input DNA compared with control ChIP experiment. (b) H3K27ac on viral LANA was measured with ChIP-qPCR assay. (c) H3K27ac on viral TR was measured with ChIP-qPCR assay. (d-g) Knockdown of SMC6 increases the levels of H3K27ac on KSHV genome. (d) iSLK.RGB cells were transduced with lentivirus expressing shRNA against SMC6. The knockdown efficiency was determined by western blots. (e-g) H3K27ac on viral RTA, LANA and TR were measured with ChIP-qPCR assay. ChIP was performed among latently infected control cells and SMC6 knockdown cells by using antibodies against H3 or H3K27ac. The recruitment of H3 and H3K27ac on KSHV genome were tested via qPCR. Representative results from three biological replicates are presented. Error bars indicate SD. Data were analyzed with Student’s t tests (**p< 0.01, ***p< 0.001).

The above evidence demonstrated that the SMC5/6 complex restricts KSHV gene transcription, and we speculated that this results from SMC5/6-mediated viral chromatin modification. To test this hypothesis, we performed ChIP on SMC6-overexpressing cells and control cells to assess the changes in several promoters of viral genes (including RTA, LANA and TR) via qPCR. Since changes in H3 occupancy may affect the enrichment of histone and acetylation modifications on the viral genome, we first examined the enrichment of total H3 on the viral genome during latency. We found no change in total histone H3 levels; however, the level of the acetylation mark H3K27ac, which is characteristic of active gene transcription, was considerably decreased in SMC6-overexpressing cells (Fig 5A–5C). Reversely, we also performed ChIP-qPCR in SMC5 or SMC6 knockdown cell line by using lentivirus-mediated shRNAs. Our results showed that the protein levels of SMC5 and SMC6 were dramatically reduced, compared to the control group (Figs 5D and S3A). Depletion of SMC5 or SMC6 increased H3K27ac histone modifications on the promoter regions of KSHV, such as LANA, RTA and TR, instead of total H3 level (Figs 5E–5G and S3B–S3D). Taken together, these results demonstrated that the SMC5/6 complex leads to a loss of H3K27ac on KSHV chromatin and forms a repressive chromatin environment.

### The SMC5/6 complex condenses KSHV chromatin

It has been shown that cohesion and condensin can compact nucleosome-bound DNA by loop extrusion [37]. We speculated that the SMC5/6 complex may also silence KSHV gene transcription through chromatin compaction. We applied an assay for transposase accessible chromatin with high-throughput sequencing (ATAC-seq) to assess KSHV chromatin accessibility. The results showed that >1×10^5^ sequencing reads mapped to the KSHV genome (S1 Table). To assess the effect of the SMC5/6 complex on viral chromatin accessibility, we performed a differential analysis of the ATAC-seq peaks between the two groups mapped to the entire length of the viral genome and found that ectopic expression of SMC6 caused a slight reduction in chromatin accessibility of the whole viral genome compared with that in the control group (S4A Fig). This may be due to the ATAC-seq results, in which most cells were latently infected by KSHV and in which most of the viral chromatin was in a silenced state. Therefore, we further analyzed the difference in the ATAC-seq peaks mapped to the latent genes and found the peaks of ORF71 in iSLK.RGB-SMC6 cells were reduced by 24% (S4B Fig), the peak of ORF72 was reduced by 22% (S4C Fig), and the peak of ORF73 was reduced by 19% (S4D Fig). This is consistent with the results that SMC6 decreases the mRNA expression of latent genes in Fig 3C. These results suggest that SMC5/6 can compact the KSHV genome and help establish a repressive chromatin structure to silence viral gene expression.

### RTA targets the SMC5/6 complex for ubiquitination and proteasomal degradation

**Fig 6:**
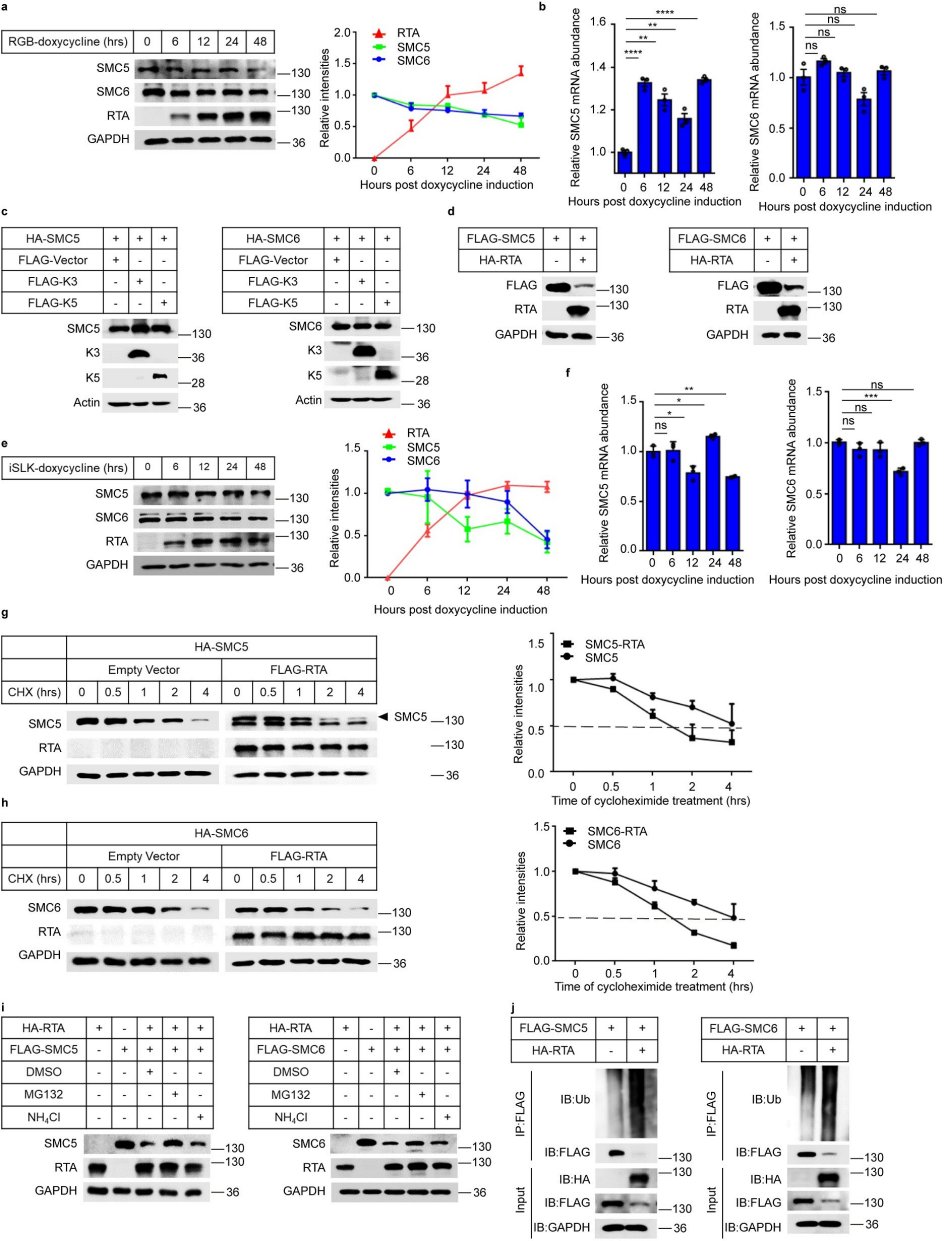
RTA targets the SMC5/6 complex for ubiquitination and proteasomal degradation. (a-b) The kinetics of SMC5 and SMC6 expression in iSLK.RGB cells. (a) iSLK.RGB cells were induced with Dox for indicated times. Cells were collected and SMC5, SMC6 and RTA expression were detected using western bolts. (b) The mRNA of SMC5 and SMC6 during the induction of lytic reactivation in iSLK.RGB cells were analyzed via qPCR. (c) KSHV K3 and K5 do not alter the expression of SMC5 and SMC6. HEK293T cells were transfected with SMC5/6 alone or together with K3 and K5. After 36h transfection, the cells were lysed and analyzed by western blotting with indicated antibodies. (d) RTA degrades SMC5 and SMC6. HEK293T cells were transfected SMC5/6 alone or together with RTA. After 36h transfection, the cells were lysed and analyzed by western blots with indicated antibodies. (e-f) The kinetics of SMC5 and SMC6 expression in iSLK.Puro cells. (e) iSLK.Puro cells were induced with Dox for indicated times. Cells were collected and SMC5, SMC6 and RTA expression were detected using western bolts. (f) The mRNA of SMC5 and SMC6 during the induction of lytic reactivation in iSLK.Puro cells were analyzed via qPCR. (g-h) RTA destabilizes the SMC5/6 complex. HEK293T cells were transfected with HA-SMC5/SMC6 alone or together with FLAG-RTA. 20h-post transfection, the cells were incubated with 100μg/ml CHX for different time points, then lysed and analyzed by immunoblotting with indicated antibodies. The relative protein abundances of RTA from immunoblots (g and h) were quantified by band intensities and normalized to the GAPDH level. (i) RTA degrades the SMC5/6 complex in a proteasome-dependent manner. HEK293T cells were transfected with HA-RTA alone or FLAG-SMC5/6 alone or HA-RTA together with FLAG-SMC5/6. 24h post-transfection, the cells transfected with HA-RTA and FLAG-SMC5/6 were treated with DMSO, MG132 and NH_4_Cl respectively for another 10h followed by detecting with western blots. (j) RTA promotes the SMC5/6 complex ubiquitination degradation. HEK293T cells were transiently transfected with FLAG-SMC5/6 alone or together with HA-RTA. After 30h transfection, the cells were treated with MG132 (10μM) for another 10h followed by immunoprecipitated with anti-FLAG antibody and then analyzed by immunoblotting with anti-ubiquitin antibodies. Representative results from three biological replicates are presented. Error bars indicate SD. Data were analyzed with Student’s multiple t tests (*p< 0.05, **p< 0.01, ***p< 0.001, ****p< 0.0001).

To explore whether KSHV adopts some strategies to resist the transcriptional inhibition of the SMC5/6 complex, we first analyzed the expression kinetics of SMC5 and SMC6 in iSLK.RGB cells during lytic reactivation. Upon Dox induction, RTA expression was increased; however, the levels of SMC5 and SMC6 were decreased (Fig 6A). We also measured the mRNA of SMC5 and SMC6 during the induction of viral lytic reactivation and found a slight increase for SMC5 but no significant change for SMC6 (Fig 6B). Therefore, we speculated that KSHV may antagonize the function of SMC5/6 by destabilizing the complex. KSHV encodes two RING-CH-containing (MARCH) family E3 ubiquitin ligases, K3 and K5, that have been shown to decrease the accumulation a variety of proteins through proteasome-mediated degradation [38–43]. In addition, the switch molecule RTA also has ubiquitin E3 ligase activity that targets multiple proteins for proteosome-mediated degradation [12,14,17,44–47]. To identify which E3 ligase of KSHV can decrease the SMC5/6 complex, we transfected plasmids expressing the SMC5/6 complex along with K3, K5 or RTA and found that K3 and K5 had no effect on SMC5/6, while RTA significantly degraded the SMC5/6 complex (Figs 6C and 6D and S5). To confirm the phenomenon of RTA-induced SMC5/6 reduction, we further analyzed the kinetics of SMC5/6 expression in iSLK.Puro (KSHV-negative) cells that stably express Dox-inducible RTA. Upon the induction of Dox, we found that with the increase in RTA, the expression of SMC5 and SMC6 was also decreased (Fig 6E), while the mRNA of SMC5 and SMC6 showed no significant change in iSLK.Puro cells (Fig 6F). Moreover, the cycloheximide (CHX)-chase half-life results showed that RTA reduced the half-life of both SMC5 and SMC6 (Fig 6G and 6H). To further explore whether RTA degrades the SMC5/6 complex through the proteasome or lysosomal pathways, we analyzed the effect of the proteasome inhibitor MG132 and lysosome inhibitor NH_4_Cl on rescuing the degradation of SMC5 or SMC6. As shown in Fig 6I, in the presence of RTA, the expression of SMC5 and SMC6 was significantly decreased; however, upon treatment with MG132, the levels of SMC5 and SMC6 displayed an obvious increase. NH_4_Cl could not rescue the degradation of the SMC5/6 complex. These results suggested that RTA degrades the SMC5/6 complex in a proteasome-dependent manner. Finally, to determine whether RTA promotes SMC5/6 degradation by catalyzing their polyubiquitylation, we assessed the effect of RTA on the ubiquitination of SMC5/6. The results showed that coexpression of RTA with SMC5 or SMC6 led to a significant increase in the ubiquitin levels of SMC5 and SMC6 compared with those in the control group (Fig 6J). Taken together, these results demonstrated that RTA targets the SMC5/6 complex for ubiquitination and proteasomal degradation.

### RTA interacts with the SMC5/6 complex

**Fig 7:**
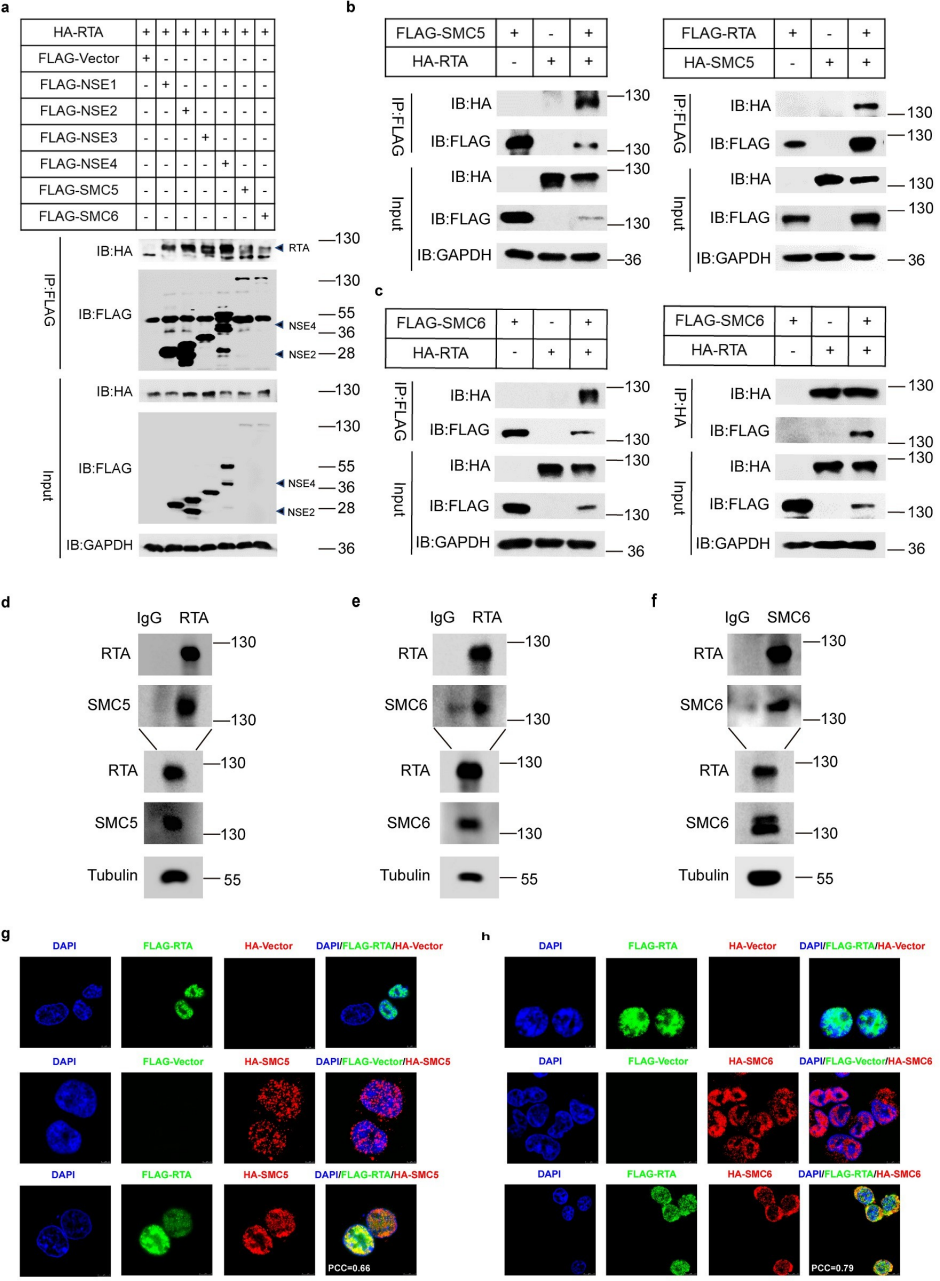
RTA interacts with the SMC5/6 complex. (a) RTA interacts with the SMC5/6 complex. HEK293T cells were transfected with HA-RTA along with FLAG-Vector or all proteins of the SMC5/6 complex. 40h post-transfection, cell lysates were collected and immunoprecipitated with anti-FLAG antibody followed by analyzing with the indicated antibodies. (b) Interaction between SMC5 and RTA. Left panel, HEK293T cells were transfected with HA-RTA alone or FLAG-SMC5 alone or HA-RTA together with FLAG-SMC5. 40h post-transfection, cell lysates were immunoprecipitated with anti-FLAG antibody and analyzed by indicated antibodies. Right panel, HEK293T cells were transfected with FLAG-RTA alone or HA-SMC5 alone or FLAG-RTA together with HA-SMC5. 40h post-transfection, cell lysates were immunoprecipitated with anti-FLAG antibody followed by analyzing with the indicated antibodies. (c) Interaction between SMC6 and RTA. HEK293T cells were transfected with HA-RTA alone or FLAG-SMC6 alone or HA-RTA together with FLAG-SMC6. 40h post-transfection, cell lysates were immunoprecipitated with anti-FLAG antibody (left) or anti-HA antibody (right) and then analyzed by indicated antibodies. (d-f) Co-IP of RTA with endogenous SMC5/6 in iSLK.RGB cells. Lytic replication of KSHV in iSLK.RGB cells was induced by Dox, and cell lysates were subjected to immunoprecipitation with anti-RTA antibody or IgG controls (d and e), anti-SMC6 antibody or IgG control (f) and analyzed by western blotting with the indicated antibodies. (g) Colocalization of SMC5 and RTA in HEK293T cells. After transfection with FLAG-RTA alone, HA-SMC5 alone or FLAG-RTA together with HA-SMC5, cells were fixed, permeated and stained with anti-HA and anti-FLAG antibody followed by incubating with second antibodies-Alexa fluor 488 and Alexa fluor 555. Colocalization was viewed by a DM6000B fluorescence microscope. Colocalization analysis was performed using Image J software. (h) Colocalization of SMC6 and RTA in HEK293T cells.

To determine whether RTA degrades the SMC5/6 complex by directly binding to those proteins, coimmunoprecipitation experiment was performed. The results showed that RTA was coprecipitated by the SMC5/6 complex (SMC5, SMC6, and NSE1-4) (Fig 7A). Given that SMC5 and SMC6 are the core heterodimers of the complex, the interactions between SMC5 and RTA and between SMC6 and RTA were further confirmed (Fig 7B and 7C). To confirm the interaction between endogenous SMC5/6 and RTA, we performed Co-IP in iSLK.RGB cells. After 24h induction by Dox to activate the expression of RTA, cell lysates were collected and immunoprecipitated with anti-RTA antibody or IgG control. As expected, endogenous SMC5 or SMC6 protein can be immunoprecipitated by RTA (Fig 7D and 7E). Since anti-SMC5 antibody is not suitable for Co-IP experiment, we only performed this experiment by using anti-SMC6 antibody. As shown in Fig 7F, RTA was associated with the endogenous SMC6 protein. We also mapped the interaction domain of RTA with SMC5/6 (S6A and S6B Fig) and determined the degradation of SMC5/6 by wild type RTA or RTA truncations (S6C and S6D Fig). Moreover, the distribution and association of RTA with SMC5 and SMC6 were examined via confocal microscopy. As shown in Fig 7G and 7H, exogenously transfected SMC5, SMC6 and RTA were mainly located in the nucleus, and upon co-transfection, they displayed obvious colocalization there. In addition, the relevance of RTA and SMC5 or SMC6 were also quantified by colocalization analysis. These results revealed that RTA interacts with the SMC5/6 complex.

### RTA-induced degradation of the SMC5/6 complex is evolutionarily conserved in γ-herpesviruses

**Fig 8:**
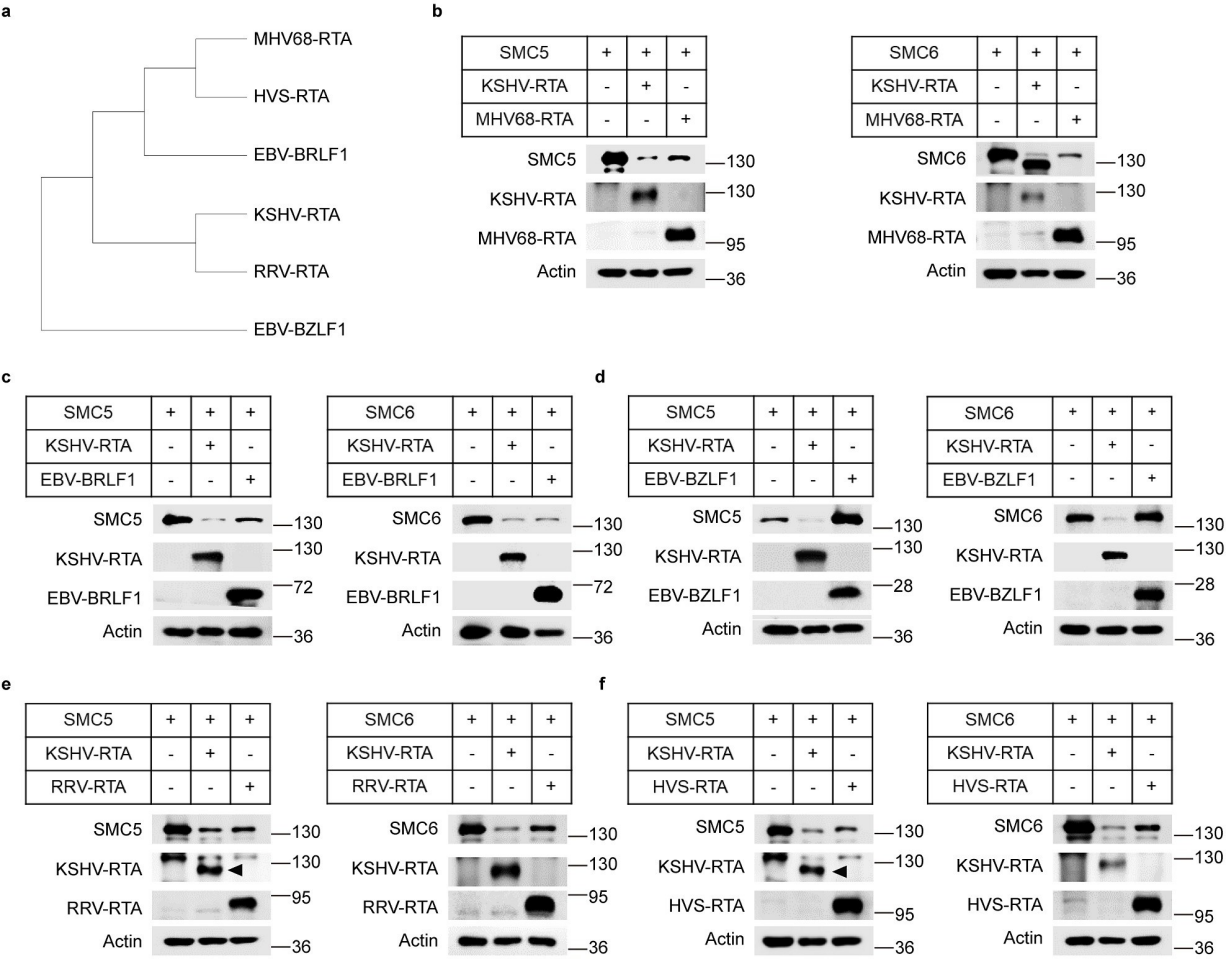
RTA-induced degradation of the SMC5/6 complex is evolutionarily conserved in γ-herpesviruses. (a) Phylogenetic analysis of the RTA proteins of γ-herpesviruses and EBV transcription-activator BZLF1. Phylogenetic analysis was performed using all amino acid sequences alignment obtained from ClustalX and the tree was built with MEGA_X_10.2.6. (b) MHV68-RTA degrades SMC5 (left) and SMC6 (right). HEK293T cells were transfected with SMC5 or SMC6 alone, SMC5 or SMC6 together with KSHV-RTA, SMC5 or SMC6 together with MHV68-RTA. 36h post-transfection, the cells were lysed and detected by western blots with indicated antibodies. (c) BRLF1 of EBV degrades SMC5 (left) and SMC6 (right). (d) EBV transcription-activator BZLF1 does not degrades SMC5 (left) and SMC6 (right). (e) RTA of RRV degrades SMC5 (left) and SMC6 (right). (f) RTA of HVS degrades SMC5 (left) and SMC6 (right).

The RTA protein is conserved among γ-herpesviruses. This protein shares a common function in transcriptional activation [48]. Through phylogenetic and positive selection analyses of the SMC5/6 complex subunits, it was found that the complex is conserved in primates and mammals [49]. To determine whether the capacity of RTA to degrade the SMC5/6 complex is a conserved function of γ-herpesviruses, we performed an evolutionary analysis among RTA proteins of the KSHV, human Epstein–Barr virus (EBV), nonhuman primate rhesus rhadinovirus (RRV), herpesvirus saimiri (HVS), and murine γ-herpesvirus 68 (MHV68). EBV BZLF1 is another transcriptional activator of EBV lacking E3 ligase activity and served as a negative control. As shown in Fig 8A, BZLF1 was evolutionarily distant from the other RTA proteins, and the other proteins were highly homologous to each other. We next determined whether these RTA homologs can degrade SMC5/6 and found that BZLF1 has no effect on SMC5 or SMC6, while RTA proteins from human, monkey and mouse γ-herpesviruses could significantly decrease SMC5 and SMC6 protein levels (Fig 8B–8F).

## Discussion

Viral replication is determined by an interaction network consisting of several key players: host factors, viral proteins and viral genomes. Although some host factors hindering KSHV replication have been identified, such as chromatin-organizing factors CTCF and cohesion [50,51], there are still many other important host factors that have not been discovered. This study revealed a new host factor, the SMC5/6 complex, which causes a significant decrease in KSHV gene transcription to hinder viral replication. Although the SMC5/6 complex is known for its silencing ability in HBV transcription, a recent study revealed that SMC5/6 interacts with the papillomavirus E2 protein and plays an essential role in viral genome maintenance [24]. These observations suggest that the functions of the SMC5/6 might be different among different viruses. Our data showed that the SMC5/6 complex inhibits KSHV replication by silencing viral gene transcription. We further studied the regulatory mechanism of the SMC5/6 complex for silencing KSHV gene transcription and found that SMC5/6 reduces the levels of the active histone mark H3K27ac on the promoters of latent LANA and TR and lytic RTA and therefore establishes a repressive chromatin structure. Recent studies have shown that lytic KSHV gene expression is under the control of both activating and repressing histone modifications [52]. The chromatin bivalency of the RTA promoter ensures the repression of RTA during latency and promotes its rapid activation upon reactivation [53]. The reduction of H3K27ac on the RTA promoter by SMC6 promotes the repression of RTA during latency, indicating that SMC5/6 may play an important role in maintaining latent infection. Histone modifications are often associated with a distinct chromatin structure and function [54]. The reduction of the active histone mark H3K27ac on KSHV genome indicates a repressive chromatin state. It has been suggested that purified human SMC5/6 complex can recognize DNA with unusual features and is capable of local DNA compaction, uncovering a possible mode for compacting extrachromosomal viral DNA [55]. However, the effect of the SMC5/6 complex on the DNA virus genome is still unclear. Our ATAC-seq results suggest that SMC6 significantly reduces the chromatin accessibility of the KSHV latent genome, indicating that SMC5/6 condenses KSHV episomes in a way similar to that of chromosome compaction. Moreover, changes in chromatin accessibility are consistent with a functionally relevant effect on silencing latent gene transcription. Interestingly, there happens to be a similar study. SLF2 condenses and silences unintegrated HIV-1 DNA, and they suggest that SLF2 inhibits unintegrated HIV-1 DNA transcription by recruiting the SMC5/6 complex [26]. However, direct evidence to show the relationship between SMC5/6 and compaction of unintegrated HIV-1 DNA is lacking. Our work reveals that the SMC5/6 complex can bind to the KSHV genome and compact genomic chromatin, and a possible working model can be proposed: the SMC5/6 complex captures KSHV genomic DNA, loads the DNA into its ring structure and condenses the genomic DNA in a way similar to that of the DNA-loop extrusion of condensin [56]. It has been suggested that the SMC6 and NSE1/3/4 subcomplexes are critical for DNA binding, and NSE3 DNA-binding mutants were shown to reduce the association of fission yeast SMC5/6 with chromatin [34,57]. Mutations in DNA binding (K220E and R229E of NSE3, G103T of SMC6) significantly increased the activation of the LANA promoter and EF1α promoter. These results convincingly suggest that the SMC5/6 complex directly associates with the KSHV genome. Several studies suggest that ATPase activity is critical for the dynamic association of SMC complexes with DNA [58]. After binding to ATP, the ATPase heads and the arms display an open conformation; upon ATP hydrolysis, the conformation of the heads change, resulting in the closure of the arm space between the two SMC subunits [59]. ATPase activity promotes conformational changes of SMC complexes and thus enables the SMC complexes to contact different DNA binding interfaces, gradually expand loops and drive loop extrusion [60]. Mutations of ATPase of SMC5 (K86I) and SMC6 (K66E) promote the activation of the KSHV LANA promoter and cellular EF1α promoter, suggesting that ATPase activity is important for promoting the formation of KSHV chromatin loops and driving loop extrusion. In addition, the SUMOylation activity of human NSE2 has been demonstrated to target Top2α, and this modification is critical for Top2α’s role in chromosome segregation [61]. It is unclear whether the SUMOylation activity of SMC5/6 is required for inhibiting transcription. Mutations of SUMOylation of NSE2 (H187A and C215A) have no effect on EF1α promoter activation. However, mutation H187A increases the activation of the LANA promoter, indicating that SUMOylation activity may not be necessary for transcription silencing. Overall, we present a model in which the SMC5/6 complex condenses the KSHV genome in a manner similar to that of DNA-loop extrusion and establishes a repressive chromatin structure by silencing viral gene expression. This work provides a new perspective for understanding gene silencing and uncovers a new therapeutic clue for viruses with episomal DNA genomes.

If a host restriction factor is a constant threat to virus replication, then the virus will inevitably evolve an equally potent counterrestriction mechanism to survive. Viral antagonists can overcome host restriction factors using various mechanisms, including coupling the restriction factors on protein degradation pathways, causing the mis-localization of the restriction factor and thus downregulating functional expression, or functioning as mimics of the restriction factor substrate [62]. Our data show that RTA can interact with the SMC5/6 complex and cause the degradation of the SMC5/6 complex through the proteasome pathway. It has been proven that the Vpu protein of HIV-1 alters the normal subcellular structure of tetherin and makes it unable to restrict viral budding, thereby antagonizing the restriction of tetherin [63]. However, the interaction between RTA and the SMC5/6 complex does not lead to mislocalization of SMC5 or SMC6, indicating that KSHV antagonizes SMC5/6 restriction mainly through the protein degradation pathway. Phylogenetic and positive selection analyses of the SMC5/6 complex indicate that the complex is conserved in primates and mammals [49]. This finding reinforces that the SMC5/6 complex may have a broad-spectrum antiviral effect on γ-herpesviruses. Accordingly, we were interested in whether RTA proteins of γ-herpesviruses can antagonize the SMC5/6 complex like RTA does of KSHV. Our study reveals that RTA-induced degradation of the SMC5/6 complex is evolutionarily conserved during γ-herpesvirus infection.

However, our study still has some limitations. We only performed ChIP-qPCR in latently infected iSLK.RGB cells to confirm the binding of SMC6 on KSHV genome, while the binding of SMC6 on KSHV genome during lytic replication has not been examined. It is important to show biological relevance of SMC5/6 association with KSHV genome in the both latent and lytic life cycles. It is worthwhile to further study in the future. In addition, it is also reasonable to perform ChIP-qPCR in other KSHV-positive cell lines, such as BCBL1, to reveal whether the binding of SMC6 on KSHV genome differs between cell lines. Importantly, the SMC5/6 complex is a dynamic machinery that undergoes conformational changes related to functions. Therefore, structural analysis of dynamic states is helpful to understand how the SMC5/6 complex binds to the KSHV genome and condenses episomes. Furthermore, future work may also need to identify the ubiquitination sites of the SMC5/6 complex.

**Fig 9:**
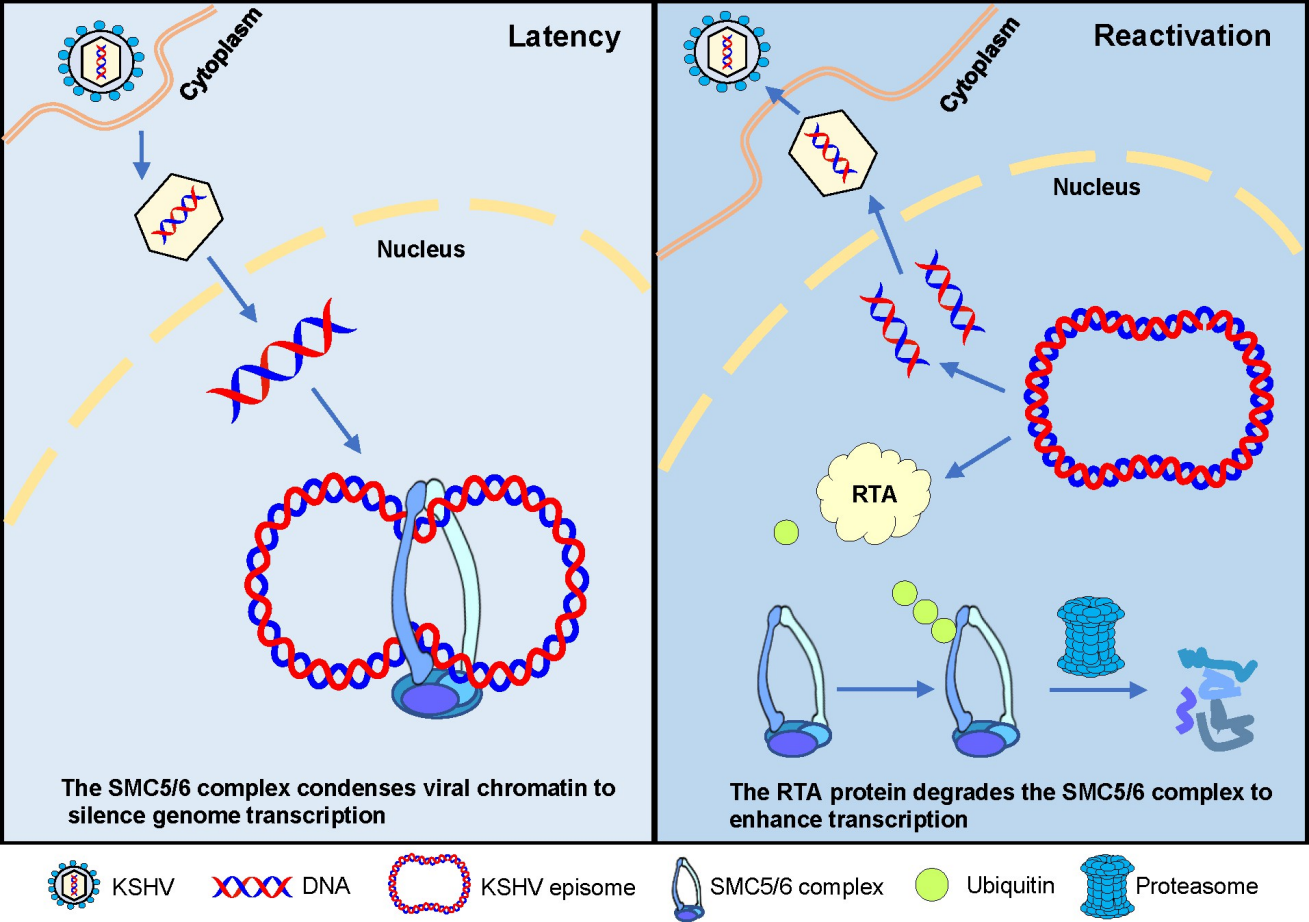
Model for the role of the SMC5/6 complex in regulating KSHV latency and reactivation. During KSHV latent replication, the host protein SMC5/6 complex binds to the KSHV episome and condenses viral chromatin, creating a repressive chromatin structure to silence genome transcription. During KSHV lytic replication, the RTA protein interacts with the SMC5/6 complex and induces ubiquitin-proteasome-mediated degradation of the SMC5/6 complex.

In summary, the current evidence supports a model for SMC5/6 complex restriction, in which the complex is associated with the KSHV genome and causes a compaction on viral chromatin, forming a repressive chromatin structure, which in turn silences viral gene expression. To counter this host defense, RTA degrades the SMC5/6 complex through the proteasome pathway, thereby promoting KSHV reactivation (Fig 9). Our work proposes a model for SMC5/6 compacting KSHV episomes and inhibiting gene transcription, which reveals a possible mechanism and provides an example for studying other extrachromosomal DNA viruses, such as HBV.

## Materials and methods

### Cell culture

iSLK.RGB cells (a kind gift from Dr. Jae Jung) were cultured in Dulbecco’s modified Eagle’s medium (DMEM, HyClone) supplemented with 10% FBS (Biological Industries), 1% antibiotics (penicillin and streptomycin, Gibco), 10μg/ml puromycin (Sigma), 250μg/ml G418 (Sigma), and 250μg/ml hygromycin B (Roche). KSHV-positive B lymphoma cell lines (BCBL1) were cultured in RPMI 1640 (HyClone) containing 10% FBS and 1% antibiotics. Human embryonic kidney (HEK) 293T cell line was maintained in DMEM supplemented with 10% FBS and 1% antibiotics. iSLK.RGB-SMC5, iSLK.RGB-SMC6, iSLK.RGB-Vector, iSLK.RGB-shSMC6, iSLK.RGB-shSMC5, and iSLK.RGB-shControl were generated by lentivirus mediated transduction according to the manufacturer’s instructions (System Bioscience, Palo Alto, USA) and were maintained in selection media with blasticidin (50μg/ml). All cell lines were grown at 37°C and 5% CO2. iSLK.RGB cells were induced by Dox or Dox together with NaB, and BCBL1 cells were induced by TPA or TPA together with NaB. Dox, TPA and NaB were purchased from Sigma-Aldrich (St. Louis, MO).

### Plasmid constructs and site-directed mutagenesis

The following plasmids were generated by PCR amplification and subcloned into the pCDH-Flag vector at Xho I and BamH I sites: pCDH-Flag-NSE1, pCDH-Flag-NSE2, pCDH-Flag-NSE3, pCDH-Flag-NSE4, pCDH-Flag-SMC5, pCDH-Flag-SMC6. The following plasmids were produced by PCR amplification and subcloned into the pCMV-HA vector at EcoR I and Xho I sites: pCMV-HA-SMC5, pCMV-HA-SMC6. Site-directed mutants pCMV-HA-SMC5-K86I and pCMV-HA-SMC6-K66E containing ATPase activity, and pCMV-HA-SMC6-G103T containing DNA binding activity were generated by PCR amplification using specific mutation primers followed by Dpn I digestion. pCDH-Flag-NSE2-H187A, pCDH-Flag-NSE2-C215A, pCDH-Flag-NSE3-K220E and pCDH-Flag-NSE3-R229E were produced by PCR amplification and Dpn I digestion. All the primers used for PCR amplification are listed in S2 Table.

### Transfection and RNAi interference, CHX and MG132 treatment

For transfection assays, HEK293T cells were plated to 70–80% confluency followed by transfecting them with the indicated expression plasmids and PEI transfection reagent, mixing thoroughly and incubating it for 15 minutes at room temperature. The mixtures were then added onto HEK293T cells and incubated for 8 h at 37°C with 5% CO2 before changing the medium to remove PEI.

For RNAi interference, iSLK-RGB cells growing in a 12-well plate (2×10^5^ cells/well) were transfected with a negative control siRNA and two siRNAs targeting human SMC6, 24h post-transfection, the second round of siRNA transfections were performed. Another 24h later, the cells were induced for KSHV lytic infection with 4μg/ml Dox and 1mM sodium butyrate in 1 ml of DMEM medium. Then cell supernatants and cell pellets were collected for detection of viral DNA copies and viral gene expression at indicated time points. The sequences of siRNAs and shRNAs against human SMC5 and SMC6 were listed in S3 Table. All siRNA transfections were performed using Lipo2000 transfection reagent (Invitrogen) according to the manufacture’s instruction.

Proteasome inhibitor CHX was purchased from MedChemExpress and was used at the concentration of 100 μg/ml, MG132 was from Beyotime biotechnology and was used at the concentration of 10 μM, NH_4_Cl was purchased from Sigma and was used at the concentration of 10 mM. For CHX treatment, cells were transfected with the indicated plasmids and medium plus proper concentrations of CHX were added 20h after transfection. At the indicated time points after CHX treatment, cells were collected and subjected to immunoblot analysis. For MG132 and NH_4_Cl treatment, cells were transfected and treated with DMSO or MG132, NH_4_Cl 24h post-transfection. Cells were harvested 10h later and detected by immunoblot.

### Western blot and immunoprecipitation

For immunoprecipitation assays, transfected cells were lysed after 40h post-transfection in Western and IP lysis buffer (Beyotime Biotechnology) supplemented with 1 mM PMSF and protease inhibitor cocktail for 30 min on ice. Cell debris were removed by centrifugation at 12,000g (15 min and 4°C), and 10% of the lysates were saved for input control, and the remaining lysate was immunoprecipitated using anti-Flag M2 affinity gel by rotating overnight at 4°C. The beads were pelleted and washed once with lysis buffer, and twice with 1×PBS, and resuspended in 30 μl 2×SDS loading dye. Input control and the immunoprecipitated complexes were then resolved on SDS-PAGE and transferred onto nitrocellulose membrane (Bio-Rad Laboratories). The membranes were incubated with appropriate antibodies followed by detection with HRP-linked secondary antibodies and visualized with ECL reagents (GE).

### RNA isolation and quantitative real-time PCR

RNA was isolated by RaPure total RNA Kit (Magen) following the manufacturer’s instructions. One microgram of RNA was used for reverse transcription with gDNA wiper HiScript III RT Super Mix (Vazyme). qPCR was performed using cDNA as template for amplifying the indicated targets listed in S2 Table by using ChamQ SYBR qPCR Master Mix (Vazyme) according to the manufacturer’s instructions. Transcript levels of each gene were quantitatively assessed by comparative CT values and normalized with control groups. Fold changes were calculated using 2^−ΔΔCT^ method and the error bars represent standard deviation of three experiment replicates.

### Virion preparation, infection and flow cytometry

For KSHV virus production and infection assays, iSLK.RGB-overexpression cells and iSLK.RGB-knockdown cells in a 12-well plate (2×10^5^ cells/well) were treated with Dox (4μg/ml) and NaB (1mM) for 72h, then the supernatant of each group was collected and used to infect HEK293T cells, after centrifugal infection for 2h (37°C, 2500rpm), the medium was removed and replaced by fresh DMEM. The cells were cultured for another 24h and KSHV-infected GFP-positive cells were observed by a fluorescent microscopy and analyzed by flow cytometry.

### Confocal microscopy and BrdU

HEK293T cells were plated to coverslips in 24-well plates (1×10^5^ cells/well), 24h later, cells were transfected with the indicated expression plasmids. 36h after transfection, cells were fixed in 4% paraformaldehyde overnight at 4°C. After three washes of PBS containing 0.1% Tween20 (PBST), cells were permeabilized with 0.1% Triton X-100 for 15 min at room temperature. Subsequently, blocking was performed in 1×PBS containing 5% milk followed by washing (5 minutes each) with 1×PBST. Anti-FLAG and anti-HA antibody were diluted in primary antibody dilution buffer (1:400) and were incubated overnight at 4°C. Cells were washed with 1×PBST followed by incubated with Alexa 488 conjugated anti-mouse secondary antibody and Alexa 555 conjugated anti-rabbit secondary antibody for 1h at room temperature. After three washes of 1×PBST, DAPI staining was performed for 5 minutes at room temperature. Finally, the coverslips were washed with 1×PBST and fixed onto slides followed by visualizing and photographing with a DM6000B fluorescence microscope (Leica, Inc., Solms, Germany).

To determine the localization of endogenous SMC6 and KSHV genomic DNA, we treated BCBL1 cells with TPA for 24h followed by replacing culture medium with BrdU (BrdU, 5-bromo-2-deoxyuridine, thymidine analog, Thermo Fisher Scientific) labeling solution (DMEM containing 10μM BrdU). After incubating with BrdU at 37°C for 2 hours, cells were washed three times with PBS and fixed with 4% formaldehyde for 15 min at room temperature. Permeabilization was performed in PBS containing 0.1% Triton X-100 for 20 min at room temperature. After removing permeabilization buffer, cells were incubated with 1M HCl for 10min on ice and 2M HCl for 10min at room temperature. Cells were washed with PBS for three times and incubated with phosphate/citric acid buffer for 10min at room temperature. After washing with Triton X-100 permeabilization buffer, anti-BrdU primary antibody and anti-SMC6 primary antibody diluted in PBS were added onto the cells overnight at 4°C. The following procedures were the same as above.

### Ubiquitination assay

HEK293T cells were transfected with plasmids expressing SMC5/SMC6 together with vector or RTA, 30h post-transfection, cell culture medium was replaced with DMEM containing MG132 (10μM) for another 10h. Then cells were lysed and immunoprecipitated using anti-Flag M2 affinity gel. After incubating overnight, the pellets were subjected to SDS-PAGE and detected by immunoblotting using an anti-ubiquitin rabbit antibody (ABclonal).

### Luciferase assays

HEK293T cells were cultured in 24-well plates followed by transfecting with the indicated expression plasmids when cells were plated to 70–80% confluency. 36h post-transfection, cells were washed twice with 1× PBS and lysed with 100 μl 1× passive lysis buffer for 15min at room temperature at a highest speed. 10 μl cell lysates were used to measure luminescence activity according to the manufacture’s instruction of dual-luciferase reporter assay system (Promega), and the remaining lysates were collected to detect protein expression.

### Chromatin immunoprecipitation and quantitative real-time PCR

iSLK.RGB-SMC6-overexpressing cells, SMC5 knockdown cells or SMC6 knockdown cells were cultured in 10cm plates (1×10^7^ cells/dish), crosslinking was performed by using 0.9% formaldehyde and rotating gently at room temperature for 10min. Stop the crosslinking by adding 2.5 M glycine to a final concentration of 0.125M and continue to rock for 5min. Wash cells twice with cold PBS and treat with trypsin for 10min. After neutralizing digestion with DMEM containing 10% FBS, cells were spined down and rinsed with cold PBS. Resuspend the nuclei in 2× 400μl nuclei lysis buffer (50 mM Tri-Cl (pH8.1), 10 mM EDTA, 1% SDS) and incubate on ice for 10min, then chromatin fragmentation was performed by using sonication (our sonicator, 30W, 5s ON, 6s OFF, 23min). Spin at 14,000 rpm for 10min at 4°C and take a small aliquot of supernatant to reverse crosslinks and save as input control, the remaining supernatant was transferred to fresh tubes and diluted for 3 folds in ChIP dilution buffer (16.7 mM Tri-Cl (pH8.1), 167 mM NaCl, 1.2 mM EDTA, 1.1% Triton X-100) followed by incubating 5μg anti-Histone H3 antibody (Proteintech, 17168) and anti-histone H3K27ac antibody (Active Motif, 39085) overnight at 4°C. Add 30μl Protein A/G beads (Sigma) and rotate for 5h at 4°C. Pellet beads and wash once in low-salt buffer (20 mM Tri-Cl (pH8.1), 150 mM NaCl, 1 mM EDTA, 1% Triton X-100, 0.1% SDS, 0.1% Na-deoxycholate), once in high-salt buffer (20 mM Tri-Cl (pH8.1), 500 mM NaCl, 1 mM EDTA, 1% Triton X-100, 0.1% SDS, 0.1% Na-deoxycholate), once in LiCl buffer (10 mM Tri-Cl (pH8.1), 250 mM LiCl, 1 mM EDTA, 0.5% NP-40, 0.5% Na-Deoxycholic acid), and twice in TE buffer (10 mM Tri-Cl (pH8.1), 1 mM EDTA). Finally, elute complex from the pellet beads in 200 μl elution buffer (1% SDS, 50 mM Tris-Cl (pH 8.1), 1 mM EDTA) and perform reverse crosslinks and PCR purification. qPCR was performed with SYBR and primers listed in S2 Table.

### Assay for Transposase Accessible Chromatin with high-throughput sequencing (ATAC-seq)

ATAC-seq was performed according to the previously described [64]. Briefly, 5×10^4^ iSLK.RGB cells were collected and immediately grinded in 2 ml of pre-chilled lysis buffer (15 mM Tris-HCl (pH7.5), 20 mM NaCl, 80 mM KCl, 0.5 mM permine, 5mM 2-ME, 0.2% TritonX-100). The total mixture was filtered with miracloth twice and then loaded on the surface of 2 ml dense sucrose buffer (20mM Tris-HCl (pH 8.0), 2 mM MgCl_2_, 2 mM EDTA, 15 mM 2-ME, 1.7M sucrose, 0.2% TritonX-100) in a 10 ml falcon tube after grinding. The nuclei were centrifuged at 2200 g at 4°C for 15 min and the pellets were resuspended in 500 μl pre-chilled lysis buffer. Mix 25 μl of reaction buffer, 2.5 μl of Nextera Tn5 Transposase, and 22.5 μl of Nuclease free H_2_O to make the transposition reaction mix. Crude nuclei were resuspended in the transposition reaction mix and incubated at 37°C for 30 min. Using a MinElute PCR Purification Kit (Qiagen,28006) to purify the DNA after transposition. The DNA was amplified using NEBNext High-Fidelity 2×PCR Master Mix for 10–15 cycles [65]. Amplified libraries were purified by using MinElute PCR Purification Kit (Qiagen,28006). The library was eluted in 20 μl Elution Buffer (10 mM Tris Buffer, pH 8.0). The quality of purified libraries was assessed using Bioanalyzer and Q-bit. The libraries were multiplexed and then sequenced using an Illumina NovaSeq 6000 with PE 150 method. For ATAC-seq data analysis, the adapter and low-quality reads were filtered out through Trimmomatic (version 0.38). Clean reads were mapped to the KSHV BAC16 genome by Hisat2 (version 2.1.0), allowing up to two mis-matches [66]. Samtools (version 1.3.1) was used to remove potential PCR duplicates, and MACS2 software (version 2.1.1.20160309) was used to call peaks by default parameters (nomodel; shift -100; extsize 200; model fold, 5, 50; q value, 0.05) [67]. The diff-peak was identified using Diffbind (version 1.16.3) [68].

### Statistical analysis

Statistical analyses were performed using Student’s t tests or multiple t tests performed in GraphPad Prism v6. Error bars represent standard deviation from triplicate samples. ns, P value >0.05; *, P value <0.05; **, P value <0.01; ***, P value < 0.001; ****, P value <0.0001.

The numerical data used in all figures are included in S1 Data.

## Supporting information

S1 Data Excel spreadsheet containing, in separate sheets, the underlying numerical data and statistical analysis for Fig 1A, 1B, 1C, 1D, 1E, 1F, Fig 1G, 1H, 2A, 2B, 2C, 2D, 2E, 2F, 2G, 2H, 3B, 3C, 3D–3F, 4B–4E, 5A–5C, 5E–5G, 6A, 6B, 6E, 6F, 6G and 6H, S2B, S2D, S3B–S3D Figs.
(XLSX)

S1 Fig Expression of SMC5 or SMC6 in SMC5/6- overexpressing cells and knockdown cells. (a-b) Expression of SMC5 or SMC6 in SMC5/6- overexpressing cells. iSLK.RGB cells overexpressing SMC5 or SMC6 were collected and the protein levels of SMC5 and SMC6 were detected by immunoblotting using antibodies against SMC5 or SMC6. (c-d) Endogenous expression of SMC5 or SMC6 in SMC5/6 knockdown cells. After 48h transfection of siRNAs, iSLK.RGB cells were collected and subjected to immunoblotting using antibodies against SMC5 or SMC6 to detect the endogenous expression of SMC5 or SMC6.
(TIF)

S2 Fig Depletion of SMC5 and SMC6 promotes KSHV replication in BCBL1 cells. (a) The knockdown efficiency of SMC5 was determined via western blots. BCBL1 cells were transfected with control siRNA and two siRNAs targeting human SMC5 for 48h post-transfection, (b) After 48h transfection of siRNAs, BCBL1 cells were induced with TPA (20ng/ml) and NaB (0.3mM) for another 36h. Then, DNase-treated viral DNAs from culture supernatants were analyzed via qPCR. Left panel, viral DNA copy numbers were quantified by using K9 primers. Right panel, pGL3-luc plasmid DNA was added during viral DNAs extraction to ensure the quality of DNA extraction. Relative DNA copy numbers were measured via qPCR using primers for K9 and pGL3. The values of control were set as 1. (c-d) Effect of knockdown SMC6 on KSHV lytic replication in BCBL1 cells, which is similar to (a-b).
(TIF)

S3 Fig Depletion of SMC5 increases H3K27ac levels on KSHV genome. (a) iSLK.RGB cells were transduced with lentivirus expressing shRNA against SMC5. The knockdown efficiency was determined by western blots. (b-d) H3K27ac on viral RTA, LANA and TR were measured with ChIP-qPCR assay. ChIP was performed among latently infected control cells and SMC5 knockdown cells by using antibodies against total H3 or H3K27ac. The recruitment of H3 and H3K27ac on KSHV genome were tested via qPCR. Data was calculated as the fold change in percentage of input DNA compared with control ChIP experiment.
(TIF)

S4 Fig The SMC5/6 complex condenses KSHV chromatin. (a) Comparing data of ATAC-seq peaks for control group with SMC6-overexpressing group. ATAC-seq was performed in latently infected control cells or SMC6-overexpressing cells. Reads were aligned to KSHV BAC-16 reference genome. To analyze the effect on chromatin accessibility, ATAC-seq peaks mapped to the entire length of the viral genome were analyzed. Reads density for the viral genome is displayed. The control group (blue) and the SMC6-overexpressing group (red) are highlighted for comparison. (b) ATAC-seq peaks mapped on latency transcript ORF71 (Gene annotation: QFU18872.1). (c) ATAC-seq peaks mapped on latency transcript ORF72 (Gene annotation: QFU18873.1). (d) ATAC-seq peaks mapped on latency transcript ORF73 (Gene annotation: QFU18874.1).
(TIF)

S5 Fig RTA degrades the SMC5/6 complex. (a) KSHV K3 and K5 do not degrade subunits NSE1-NSE4 of the SMC5/6 complex. HEK293T cells were transfected with K3 or K5 together with NSE1-NSE4. 36h post-transfection, cells were collected and western blots were performed with indicated antibodies. (b) KSHV RTA degrades subunits NSE1-NSE4 of the SMC5/6 complex. HEK293T cells were transfected with RTA together with NSE1-NSE4. 36h post-transfection, cells were collected and western blots were performed with indicated antibodies.
(TIF)

S6 Fig Mapping the interaction domain of RTA with SMC5 or SMC6. (a) Mapping the interaction domain of RTA with SMC5. Co-IP and western blotting of 293T cells transfected with HA-tagged SMC5 along with Flag-tagged RTA truncations or full-length RTA. An empty vector was used as a negative control. (b) Mapping the interaction domain of RTA with SMC6. (c) Defining the activity of RTA truncations and the full-length RTA in degradation of SMC5. HA-SMC5 and full-length RTA or RTA truncations were transfected 293T cells. 48h after transfection, cell lysates were collected and analyzed by western blot assays. (d) Defining the activity of RTA truncations and the full-length RTA in degradation of SMC6.
(TIF)

S1 Table Reads for ATAC-seq libraries. (DOCX)

S2 Table Primers for PCR amplification, qPCR and ChIP-qPCR analysis. (DOCX)

S3 Table Sequences of siRNAs and shRNAs. (DOCX)
