# Supplementary figures and images for: KSHV RTA antagonizes SMC5/6 complex-induced viral chromatin compaction by hijacking the ubiquitin-proteasome system
Source: PLoS Pathog. 2022 Aug 1;18(8):e1010744. doi: 10.1371/journal.ppat.1010744 (PMC9371351; doi:10.1371/journal.ppat.1010744)

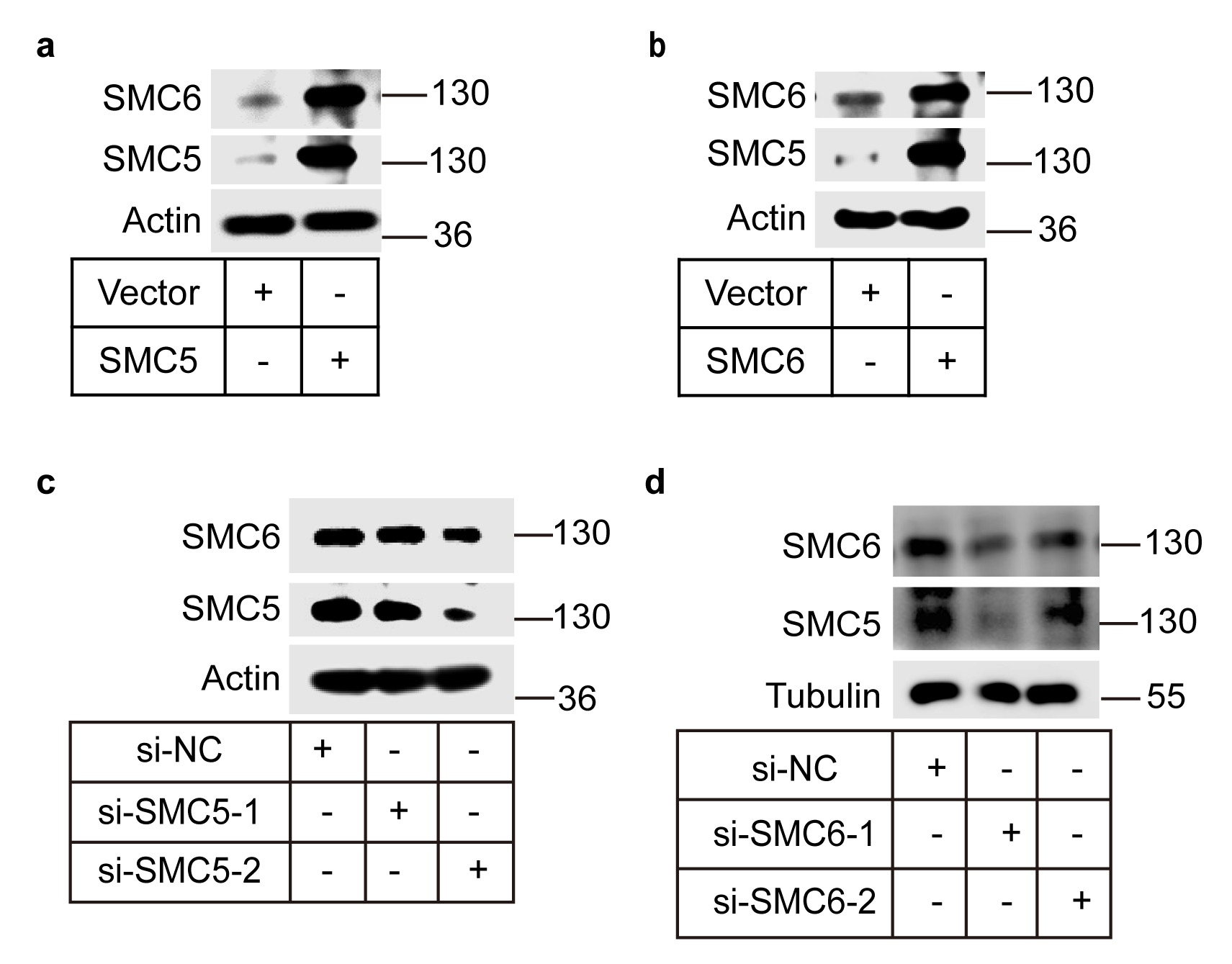

Supplement: S1 Fig — (a-b) Expression of SMC5 or SMC6 in SMC5/6- overexpressing cells. iSLK.RGB cells overexpressing SMC5 or SMC6 were collected and the protein levels of SMC5 and SMC6 were detected by immunoblotting using antibodies against SMC5 or SMC6. (c-d) Endogenous expression of SMC5 or SMC6 in SMC5/6 knockdown cells. After 48h transfection of siRNAs, iSLK.RGB cells were collected and subjected to immunoblotting using antibodies against SMC5 or SMC6 to detect the endogenous expression of SMC5 or SMC6. (TIF) [file ppat.1010744.s002.tif]

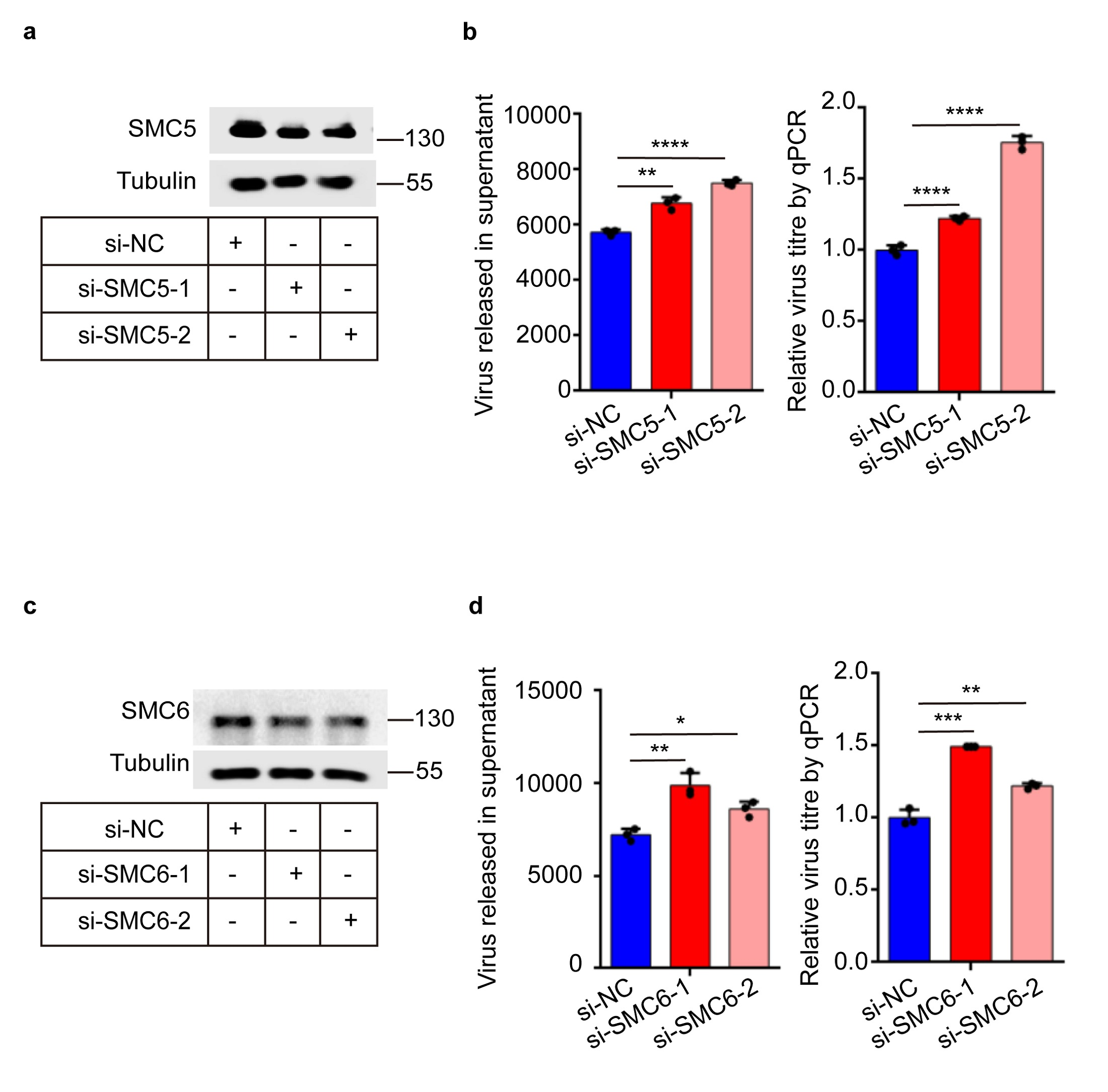

Supplement: S2 Fig — (a) The knockdown efficiency of SMC5 was determined via western blots. BCBL1 cells were transfected with control siRNA and two siRNAs targeting human SMC5 for 48h post-transfection, (b) After 48h transfection of siRNAs, BCBL1 cells were induced with TPA (20ng/ml) and NaB (0.3mM) for another 36h. Then, DNase-treated viral DNAs from culture supernatants were analyzed via qPCR. Left panel, viral DNA copy numbers were quantified by using K9 primers. Right panel, pGL3-luc plasmid DNA was added during viral DNAs extraction to ensure the quality of DNA extraction. Relative DNA copy numbers were measured via qPCR using primers for K9 and pGL3. The values of control were set as 1. (c-d) Effect of knockdown SMC6 on KSHV lytic replication in BCBL1 cells, which is similar to (a-b). (TIF) [file ppat.1010744.s003.tif]

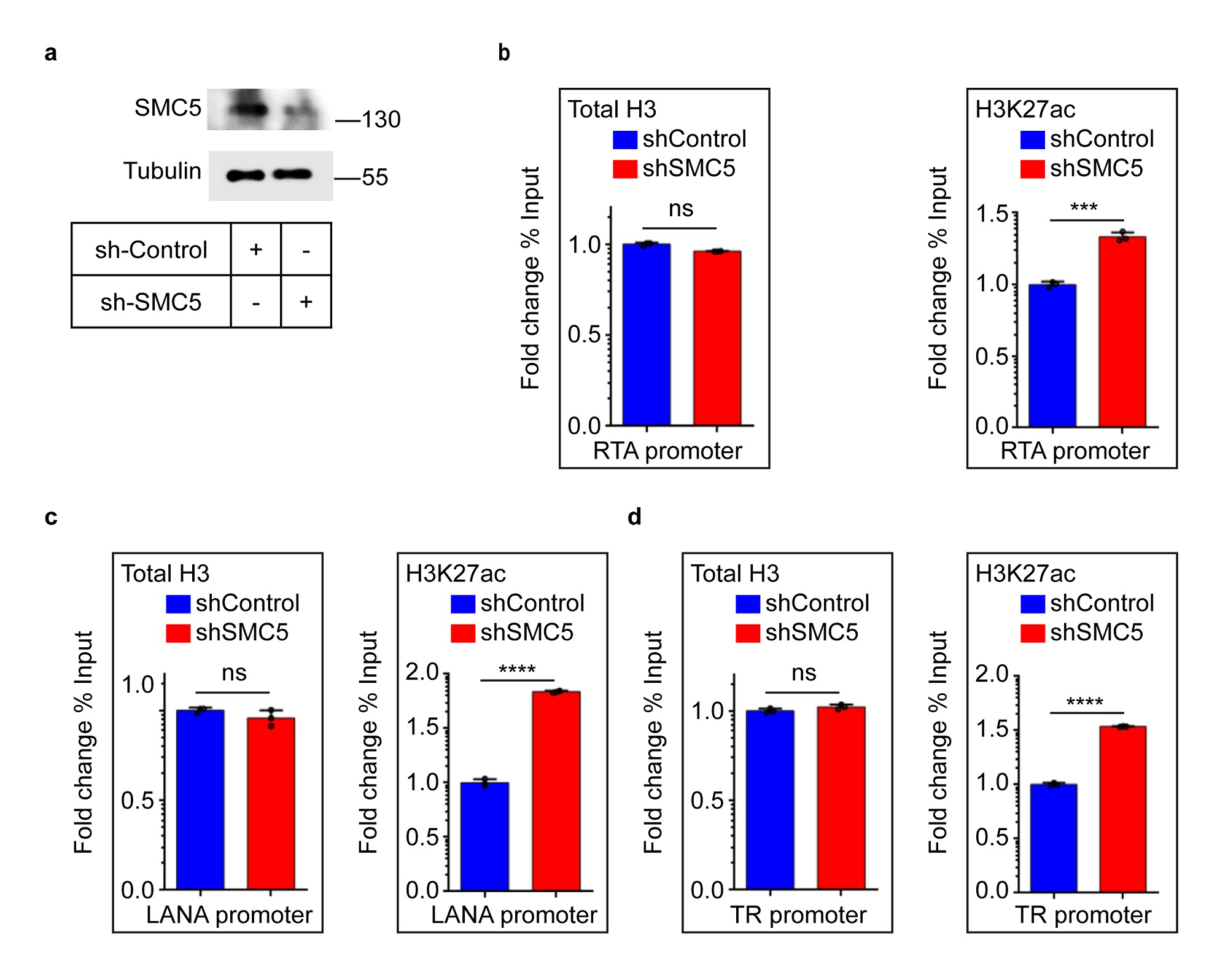

Supplement: S3 Fig — (a) iSLK.RGB cells were transduced with lentivirus expressing shRNA against SMC5. The knockdown efficiency was determined by western blots. (b-d) H3K27ac on viral RTA, LANA and TR were measured with ChIP-qPCR assay. ChIP was performed among latently infected control cells and SMC5 knockdown cells by using antibodies against total H3 or H3K27ac. The recruitment of H3 and H3K27ac on KSHV genome were tested via qPCR. Data was calculated as the fold change in percentage of input DNA compared with control ChIP experiment. (TIF) [file ppat.1010744.s004.tif]

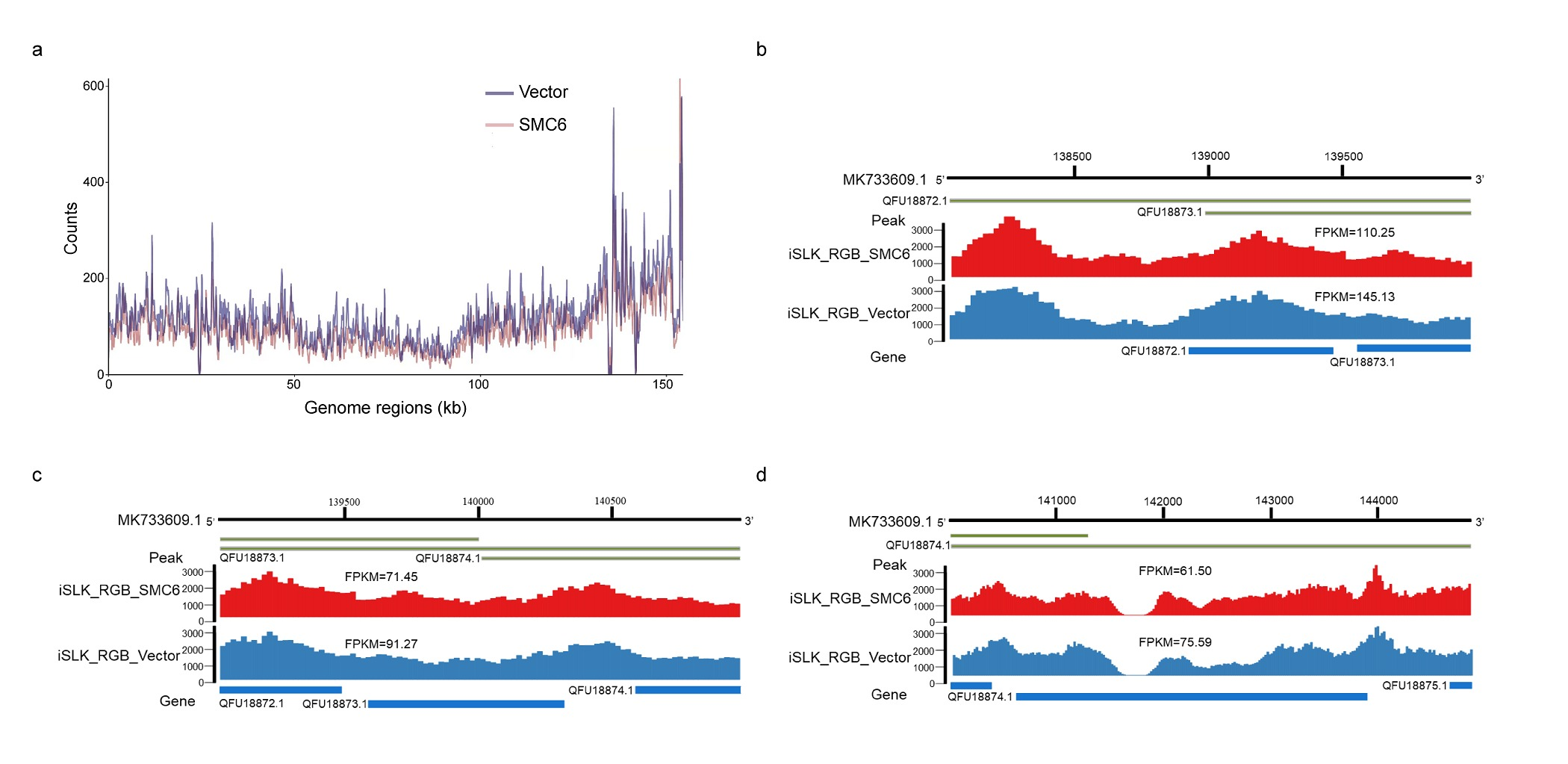

Supplement: S4 Fig — (a) Comparing data of ATAC-seq peaks for control group with SMC6-overexpressing group. ATAC-seq was performed in latently infected control cells or SMC6-overexpressing cells. Reads were aligned to KSHV BAC-16 reference genome. To analyze the effect on chromatin accessibility, ATAC-seq peaks mapped to the entire length of the viral genome were analyzed. Reads density for the viral genome is displayed. The control group (blue) and the SMC6-overexpressing group (red) are highlighted for comparison. (b) ATAC-seq peaks mapped on latency transcript ORF71 (Gene annotation: QFU18872.1). (c) ATAC-seq peaks mapped on latency transcript ORF72 (Gene annotation: QFU18873.1). (d) ATAC-seq peaks mapped on latency transcript ORF73 (Gene annotation: QFU18874.1). (TIF) [file ppat.1010744.s005.tif]

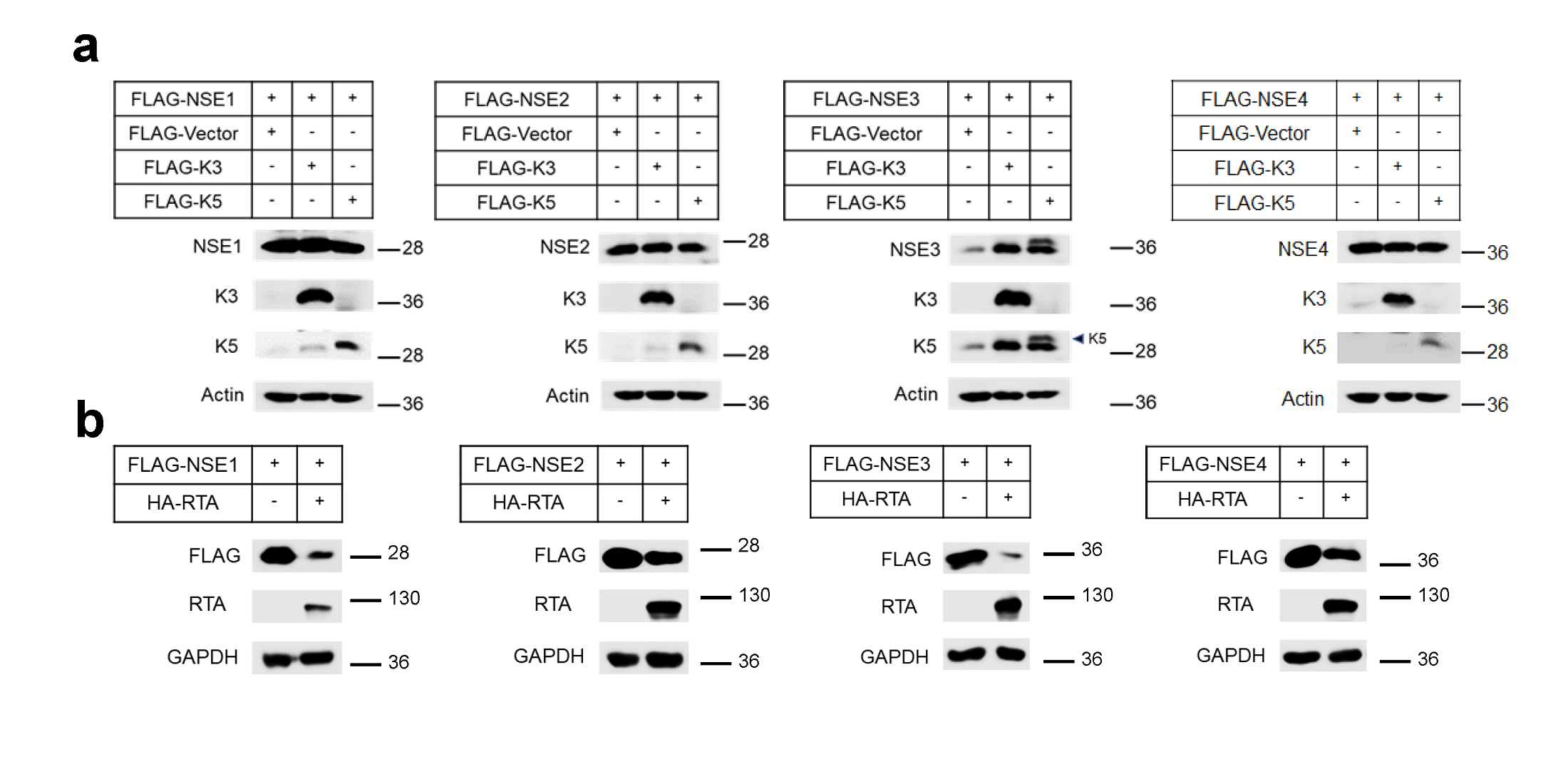

Supplement: S5 Fig — (a) KSHV K3 and K5 do not degrade subunits NSE1-NSE4 of the SMC5/6 complex. HEK293T cells were transfected with K3 or K5 together with NSE1-NSE4. 36h post-transfection, cells were collected and western blots were performed with indicated antibodies. (b) KSHV RTA degrades subunits NSE1-NSE4 of the SMC5/6 complex. HEK293T cells were transfected with RTA together with NSE1-NSE4. 36h post-transfection, cells were collected and western blots were performed with indicated antibodies. (TIF) [file ppat.1010744.s006.tif]

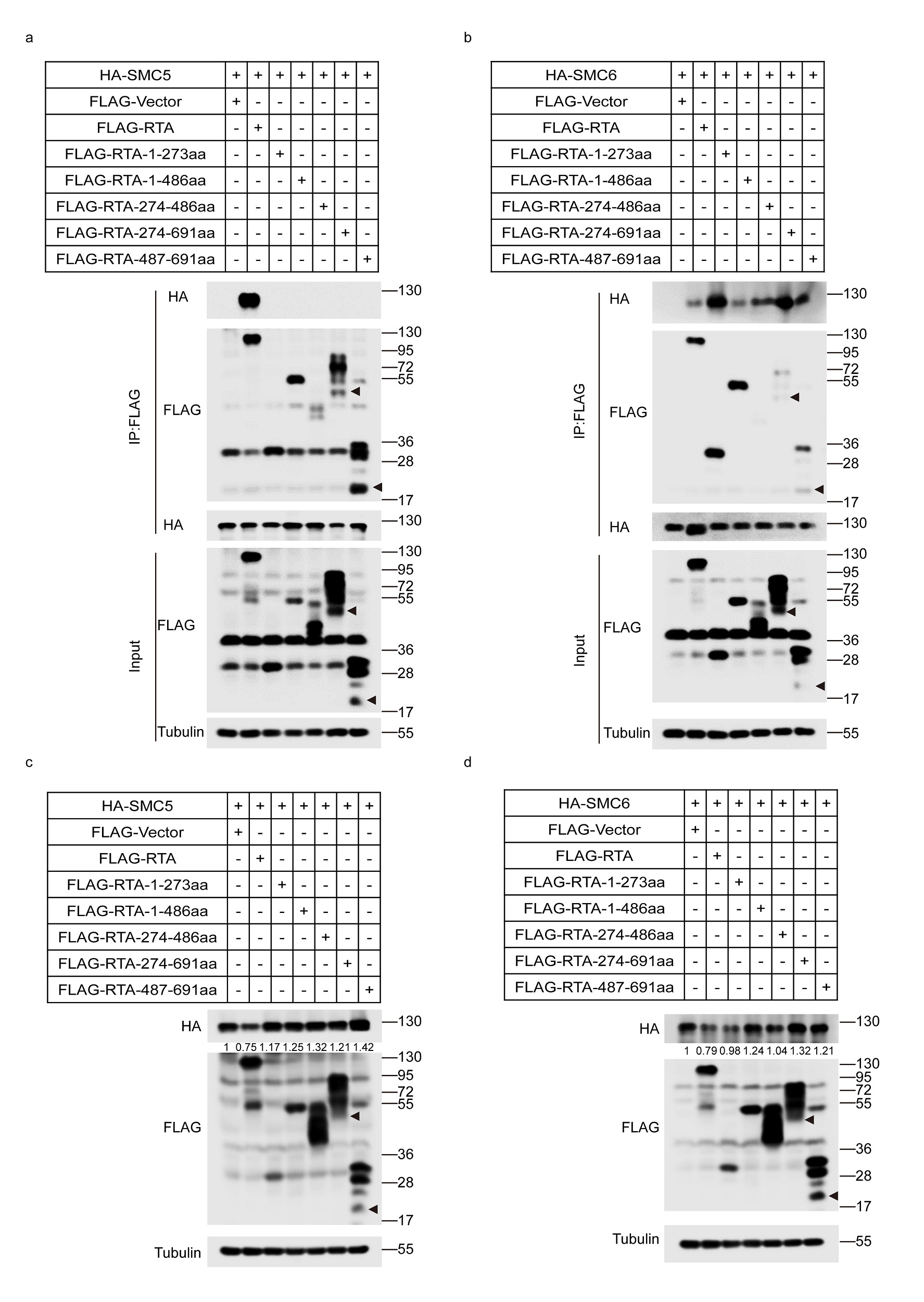

Supplement: S6 Fig — (a) Mapping the interaction domain of RTA with SMC5. Co-IP and western blotting of 293T cells transfected with HA-tagged SMC5 along with Flag-tagged RTA truncations or full-length RTA. An empty vector was used as a negative control. (b) Mapping the interaction domain of RTA with SMC6. (c) Defining the activity of RTA truncations and the full-length RTA in degradation of SMC5. HA-SMC5 and full-length RTA or RTA truncations were transfected 293T cells. 48h after transfection, cell lysates were collected and analyzed by western blot assays. (d) Defining the activity of RTA truncations and the full-length RTA in degradation of SMC6. (TIF) [file ppat.1010744.s007.tif]
