# Supplementary material for: KSHV RTA antagonizes SMC5/6 complex-induced viral chromatin compaction by hijacking the ubiquitin-proteasome system
Source: PLoS Pathog. 2022 Aug 1;18(8):e1010744. doi: 10.1371/journal.ppat.1010744 (PMC9371351; doi:10.1371/journal.ppat.1010744)
Supplement: S1 Table — (DOCX) [file ppat.1010744.s008.docx]

**S1 Table. Reads for ATAC-seq libraries**

| Sample | Number of total reads | Number of mapped reads |
| --- | --- | --- |
| iSLK.RGB-Vector-1 | 112266720 | 106729 |
| iSLK.RGB-Vector-2 | 109937934 | 101796 |
| iSLK.RGB-SMC6-1 | 84698864 | 93708 |
| iSLK.RGB-SMC6-2 | 85602776 | 79928 |
