# Supplementary material for: KSHV RTA antagonizes SMC5/6 complex-induced viral chromatin compaction by hijacking the ubiquitin-proteasome system
Source: PLoS Pathog. 2022 Aug 1;18(8):e1010744. doi: 10.1371/journal.ppat.1010744 (PMC9371351; doi:10.1371/journal.ppat.1010744)
Supplement: S2 Table — (DOCX) [file ppat.1010744.s009.docx]

**S2 Table. Primers for PCR amplification, qPCR and ChIP- qPCR analysis**

| Primer name | Sequence of oligonucleotide (5’-3’) |
| --- | --- |
| pCDH-FLAG-SMC6-F | CTAGCGCTACCGGACTCAGATCTCGAGATGGCCAAAAGAAAGGAAG |
| pCDH-FLAG-SMC6-R | TTCTCGAACTGAGGGTGGCTCCAGGATCCCCTTTGGTCATCATCTTCTTC |
| pCDH-FLAG-SMC5-F | CTAGCGCTACCGGACTCAGATCTCGAGATGGCGACTCCGAGCAAGAAG |
| pCDH-FLAG-SMC5-R | TTCTCGAACTGAGGGTGGCTCCAGGATCCAGAAGGTTGAGTGAATGTAATACG |
| pCDH-FLAG-NSE4-F | CTAGCGCTACCGGACTCAGATCTCGAGATGTCTGGGGACAGCAGCG |
| pCDH-FLAG-NSE4-R | TTCTCGAACTGAGGGTGGCTCCAGGATCCAGCACTTGGCTTCTG |
| pCDH-FLAG-NSE3-F | CTAGCGCTACCGGACTCAGATCTCGAGATGTTGCAAAAACCGAG |
| pCDH-FLAG-NSE3-R | TTCTCGAACTGAGGGTGGCTCCAGGATCCAGAGGATGGAGCTGG |
| pCDH-FLAG-NSE2-F | CTAGCGCTACCGGACTCAGATCTCGAGATGCCAGGACGTTCCAG |
| pCDH-FLAG-NSE2-R | TTCTCGAACTGAGGGTGGCTCCAGGATCCCTCGGAATGACGATGTC |
| pCDH-FLAG-NSE1-F | CTAGCGCTACCGGACTCAGATCTCGAGATGCAGGGCAGCACAAG |
| pCDH-FLAG-NSE1-R | TTCTCGAACTGAGGGTGGCTCCAGGATCCATGCTGCCTGGACCG |
| pCMV-HA-SMC5-F | CCGGAATTCCGATGGCGACTCCGAGCAAGAAG |
| pCMV-HA-SMC5-R | CCGCTCGAGCGGTCAAGAAGGTTGAGTGAATGTAATACG |
| pCMV-HA-SMC6-F | CCGGAATTCCGATGGCCAAAAGAAAGGAAG |
| pCMV-HA-SMC6-R | CCGCTCGAGTCACCTTTGGTCATCATCTTCTTC |
| pCDH-FLAG-NSE2-H187A-F | GAAAAATAAAGTGTGTGGCGCCACCTATGAAGAG |
| pCDH-FLAG-NSE2-H187A-R | GGCGCCACACACTTTATTTTTCACTGGCTTCTTC |
| pCDH-FLAG-NSE2-C215A-F | CTATTGCCCTCAAATTGGCGCTAGCCACACGGATATAAG |
| pCDH-FLAG-NSE2-C215A-R | AGCGCCAATTTGAGGGCAATAGGCCTTTTTCTTCC |
| pCDH-FLAG-NSE3-K220E-F | CATTTAATTTTCGGAGATCCAGAGAAACTCATTACTG |
| pCDH-FLAG-NSE3-K220E-R | CTCTGGATCTCCGAAAATTAAATGCTTCTTGGTGGGGTAG |
| pCDH-FLAG-NSE3-R229E-F | CATTACTGAGGACTTTGTGGAACAGCGTTACCTGG |
| pCDH-FLAG-NSE3-R229E-R | TTCCACAAAGTCCTCAGTAATGAGTTTCTTTGGATCTCCG |
| pCMV-HA-SMC5-K86I-F | GAGCCAATGGAACAGGGATTTCGAGCATTGTGTG |
| pCMV-HA-SMC5-K86I-R | AATCCCTGTTCCATTGGCTCCAACGATCATATTC |
| pCMV-HA-SMC6-K66E-F | GTCATTCAATGCTTGGACCTTTTGAGTTTGGTTCTAATG |
| pCMV-HA-SMC6-K66E-R | CTCAAAAGGTCCAAGCATTGAATGACACATGAAGTTTTTTAG |
| pCMV-HA-SMC6-G103T-F | GCAGTTGCTACTAATAGAACATCCTCTTTAAAAGG |
| pCMV-HA-SMC6-G103T-R | TGTTCTATTAGTAGCAACTGCTCTTCCACCAAGAC |
| qPCR-K9-F | GTCTCTGCGCCATTCAAAAC |
| qPCR-K9-R | CCGGACACGACAACTAAGAA |
| qPCR-RTA-F | AGACCCGGCGTTTATTAGTACGT |
| qPCR-RTA-R | CAGTAATCACGGCCCCTTGA |
| qPCR-ORF73-F | GCTTGGTCCGGCTGACTTAT |
| qPCR-ORF73-R | TGCAGTACCGCCCATGG |
| qPCR-ORF72-F | CATTGCCCGCCTCTATTATCA |
| qPCR-ORF72-R | ATGACGTTGGCAGGAACCA |
| qPCR-ORF71-F | GGATGCCCTAATGTCAATGC |
| qPCR-ORF71-R | GGCGATAGTGTTGGGAGTGT |
| qPCR-ORF59-F | TTGGCACTCCAACGAAATATTAGAA |
| qPCR-ORF59-R | CGGGAACCTTTTGCGAAGA |
| qPCR-ORF57-F | TGGACATTATGAAGGGCATCCTA |
| qPCR-ORF57-R | CGGGTTCGGACAATTGCT |
| qPCR-ORF26-F | CAGTTGAGCGTCCCAGATGA |
| qPCR-ORF26-R | GGAATACCAACAGGAGGCCG |
| qPCR-ORF25-F | CGTATCCCCTGTTCTGCTATG |
| qPCR-ORF25-R | TTTTCCCGAGTTGACCCAG |
| qPCR-Luc-F | TCGTCACATCTCATCTACC |
| qPCR-Luc-R | CTTTAGGCAGACCAGTAG |
| qPCR-GAPDH-F | GGAGCGAGATCCCTCCAAAAT |
| qPCR-GAPDH-R | GGCTGTTGTCATACTTCTCATGG |
| qPCR-SMC5-F | TCCCGAGAGACCCTTCGTC |
| qPCR-SMC5-R | TTCCATTGGCTCCAACGATCA |
| qPCR-SMC6-F | CCTAAAAATGCCAAAAGGCCAAG |
| qPCR-SMC6-R | TTTACATTCGTCTTCGTCACCAT |
| qPCR-PJA1-F | GATGGGTTAGGAGTGGCTGAA |
| qPCR-PJA1-R | ACGGCTTGTGGAAA TAGTGG |
| qPCR-18S rRNA-F | CGGCTACCACATCCAAGGAA |
| qPCR-18S rRNA-R  ChIP-qPCR-RTA-F | GCTGGAATTACCGCGGCT  TCCCCTTCTCCACCGTCA |
| ChIP-qPCR-RTA-R | TCCGCAATGTCAGGTTCCAC |
| ChIP-qPCR-LANA-F | GTTTATAAGTCAGCCGGACCAA |
| ChIP-qPCR-LANA-R | GATATAACTCCGCCCTCCACTA |
| ChIP-qPCR-TR-F | GGGGGACCCCGGGCAGCGAG |
| ChIP-qPCR-TR-R | GGCTCCCCCAAACAGGCTCA |
