# Supplementary material for: KSHV RTA antagonizes SMC5/6 complex-induced viral chromatin compaction by hijacking the ubiquitin-proteasome system
Source: PLoS Pathog. 2022 Aug 1;18(8):e1010744. doi: 10.1371/journal.ppat.1010744 (PMC9371351; doi:10.1371/journal.ppat.1010744)
Supplement: S3 Table — (DOCX) [file ppat.1010744.s010.docx]

**S3 Table. Sequences of siRNAs and shRNAs**

| Sequence name | Sequence of oligonucleotide (5’-3’) |
| --- | --- |
| siSMC5#1-sense | GCUUCUGGAAAUCUUGUAATT |
| siSMC5#1-antisense | UUACAAGAUUUCCAGAAGCTT |
| siSMC5#2-sense | GCUCGAGACCUCAUGCAAATT |
| siSMC5#2-antisense | UUUGCAUGAGGUCUCGAGCTT |
| siSMC6#1-sense | GCAACAGGCUCCGUGGUUUTT |
| siSMC6#1-antisense | AAACCACGGAGCCUGUUGCTT |
| siSMC6#2-sense | GCACCAGAAUGUAUGGCAUTT |
| siSMC6#2-antisense | AUGCCAUACAUUCUGGUGCTT |
| sh-SMC5 | GCGAAACTTGTTACCGAATTA |
| sh-SMC6 | CACCCTACCAAGAGCTTATAA |
